# FISH-FACS proteomics: enhanced label-free quantitative proteome analysis from low cell numbers of uncultured environmental microorganisms

**DOI:** 10.1093/ismeco/ycaf145

**Published:** 2025-08-23

**Authors:** Vaikhari Kale, Ga Yan Grace Ho, Sandra Maaß, Anke Trautwein-Schult, Daniel Bartosik, Thomas Schweder, Bernhard M Fuchs, Dörte Becher

**Affiliations:** Institute of Microbiology, University of Greifswald, Greifswald 17489, Germany; Max Planck Institute for Marine Microbiology, Bremen 28359, Germany; Institute of Microbiology, University of Greifswald, Greifswald 17489, Germany; Institute of Microbiology, University of Greifswald, Greifswald 17489, Germany; Institute of Pharmacy, University of Greifswald, Greifswald 17489, Germany; Institute of Marine Biotechnology e.V., Greifswald 17489, Germany; Institute of Pharmacy, University of Greifswald, Greifswald 17489, Germany; Institute of Marine Biotechnology e.V., Greifswald 17489, Germany; Max Planck Institute for Marine Microbiology, Bremen 28359, Germany; Institute of Microbiology, University of Greifswald, Greifswald 17489, Germany; Institute of Marine Biotechnology e.V., Greifswald 17489, Germany

**Keywords:** algae bloom, North Sea, bacterioplankton, FISH, FACS, proteome, low biomass

## Abstract

Metaproteomics is an essential approach to analyze the *in situ* metabolic activity of microbes across various environments. In such highly diverse environmental samples, the functionality of specific microorganisms of importance often remains underexplored due to the protein inference problem arising from sequence similarities between organisms. One approach to overcome this challenge is the enrichment of uncultured target organisms. However, this often results in samples with low protein content. In this study, we have developed a workflow that combines fluorescence *in situ* hybridization (FISH) and fluorescence-activated cell sorting (FACS) with mass spectrometry-based proteomics to analyze proteins from uncultured bacteria directly from environmental samples. We demonstrate that 1 × 10^5^ bacterial cells are sufficient for reliable qualitative protein identifications, while 5 × 10^5^ to 1 × 10^6^ cells allow for both reproducible protein identification and quantification after FISH and FACS. In addition, the use of a clade-specific database enhances data analysis by improving peptide mapping, especially when compared to metaproteomics results.

## Introduction

Proteomics has rapidly developed in recent decades, driven by technological advances, recognition of the limitations of genomics, medical demands, and an increasing focus on systems biology. Advances in mass spectrometry (MS), bioinformatics, and high-throughput techniques have paved the way for comprehensive studies of proteins and their functions in biological systems [1]. Technologically, the development of metaproteomics methods represents a consistent progression within proteomics. Metaproteomics is the large-scale study of all proteins within a microbial community or microbiome at a given time [2]. While classical proteomics focuses on the study of proteins in a single biological sample (such as a cell or tissue), metaproteomics examines the protein profiles of complex microbial communities, such as those found in soil, water, or the human gut [3–7]. These communities, which usually consist of bacteria, archaea, viruses, and eukaryotic microbes, interact with each other and influence the function of the entire community.

Metaproteomics complements the genomic blueprint and gene expression data obtained from metagenomics and metatranscriptomics by identifying the temporal and spatial abundance of metabolic enzymes and other proteins. Furthermore, metaproteomics provides insights into which specific microorganisms are responsible for particular functions within a microbial ecosystem [8]. By going beyond pure DNA analysis, metaproteomics provides crucial insights into the actual physiological activity of microorganisms in various environments, making it a valuable tool in environmental research, medicine, and biotechnology [9].

While metaproteomics is a widely used method for exploring the functional protein composition of microbial communities, it still faces several technical, analytical, and biological challenges that can affect the application and interpretation of results [10]. In bottom-up proteomics, proteins are digested into peptides, which are then matched to protein databases for identification. In this context, the high complexity of microbial communities makes metaproteomic data often difficult to interpret as many microorganisms, especially those that are closely related, share similar or even identical proteins. Consequently, only certain peptides within a protein can be unambiguously assigned to a protein from a specific organism, while others may have multiple possible origins. Conserved proteins (those found in multiple organisms and showing little variation) are widespread and complicate unique peptide and protein assignments [11]. This challenge makes it difficult to precisely determine the functional roles of individual microorganisms, often requiring additional experimental methods for more accurate assignments.

One effective strategy for analyzing the functional role of individual members of microbial communities in environmental samples is the enrichment of those uncultured key species. In this study, we present the combination of fluorescence *in situ* hybridization (FISH) with fluorescence-activated cell sorting (FACS) as a method to reduce community complexity prior to MS-based proteome analysis of a target microbial population within this community. FISH using 16S rRNA-targeted oligonucleotide probes allows the identification and enumeration of taxonomically defined clades in such complex environmental samples [12]. FISH probes have to be carefully selected to avoid broad phylogenetic target, as it may result in inadequate enrichment of the target bacterial clade cells for downstream proteomic sample preparation. The target clade is defined by the researcher and is ideally at the species or genus level, in order to leverage significant reduction in database size and the number of shared peptide sequences for downstream proteome analysis. While FACS alone has been routinely applied for downstream proteome analysis of both eukaryotic and prokaryotic cells [13–18], the combination of FISH, FACS, and proteomics on complex communities is particularly challenging due to the required MS-compatibility of fixatives and reagents necessary for FISH, limitations on sorting speed of low-abundance populations, as well as limited sample material prior to sorting. The latter challenge leads to samples with extremely low biomass, which complicates downstream proteomic sample preparation, and negatively impacts protein identification and quantification. Therefore, step-wise methods optimization of protein extraction, enzymatic digestion to peptides, and the detection method on the mass spectrometer, is essential to ensure high-quality proteome analysis.

The workflow presented here was first established and optimized using the culturable bacterial strain *Polaribacter* sp. KT25b, an isolate from the North Sea [19]. *Polaribacter* species (members of the family *Flavobacteriaceae*) serve as a model for studying the physiology of marine microbes due to their predicted ability to metabolize laminarin, a widespread energy storage polysaccharide in marine algae [20]. To ensure accurate quantification of varying protein profiles in samples processed through this workflow, we cultivated *Polaribacter* sp. KT25b using two different carbon sources and compared the protein abundance profiles before and after implementing the enrichment workflow. We used defined cell number (1 × 10^6^ and 5 × 10^5^ cells) of this model bacterium to mimic the available environmental biomass, corresponding to total protein amount of ~100 ng or less.

Finally, we applied the optimized workflow to seawater samples collected off the coast of Helgoland to verify the method’s suitability for analyzing environmental samples. For this, we looked at the response of heterotrophic bacterioplankton to marine phytoplankton blooms, which occur naturally due to seasonal changes in nutrient and light availability [21]. These microalgal blooms lead to significant carbon fixation and create conditions for rapid bacterial growth through increased dissolved organic material [22]. The key bacterioplankton groups such as *Bacteriodota*, *Gammaproteobacteria*, and *Verruccomicrobiota* thrive on specific organic substrates [23]. Bacterioplankton are adapted to utilize these organic substrates, such as phytoplankton-derived polysaccharides, through the evolution of carbohydrate-active enzymes (CAZymes) and TonB-dependent transport systems (TBDTs) [21, 24, 25]. Our focus was on *Aurantivirga*, an uncultured sister genus to *Polaribacter*, which regularly peaks in abundance and is transcriptionally active during the Helgoland spring bloom [23, 26]. Here we demonstrate that combining FISH-FACS and MS-based proteomics is a promising approach for gaining deeper insights into the *in situ* activity of key bacterioplankton during North Sea phytoplankton blooms.

## Materials and methods

### Cultivation

The strain *Polaribacter* sp. KT25b (NCBI: txid1855336) was cultivated in synthetic seawater medium (MPM) [27] supplemented with 0.2% (w/v) glucose or laminarin at 20°C shaking at 180 rpm in 300 ml alu-cap cultivation flasks. Bacterial growth was monitored with optical density measurements at 600 nm. A secondary culture was inoculated to achieve a starting OD_600_ of 0.05 using the primary culture grown from 1 ml glycerol stock. Cells were harvested after 13 h during the mid-exponential phase based on the growth curve (Supplementary Fig. S1). Cell pellets were centrifuged for 20 min at 8000 × g at 4°C and the supernatant was discarded. The pellets were washed with 1× PBS (from 10× stock: 100 mM Na_2_HPO_4_ × 2H_2_O, 18 mM KH_2_PO_4_, 1.37 M NaCl, 27 mM KCl; pH 7.4; 0.2 μm filter sterilized).

### Generation of low biomass samples from *Polaribacter* sp. KT25b

To prepare low biomass samples, cells collected during the mid-exponential phase were stained with DNA/RNA stain Syto 9 (500 nmol/l) and counted on a Guava easyCyte flow cytometer (Merck Millipore). The excitation wavelength was 488 nm and the emission was collected at 515–545 nm. Based on this count, the required culture volume to contain 1×10^6^ or 5×10^5^ cells was calculated. Five sample replicates for both, glucose and laminarin-grown cells, were collected and stored at −80°C, representing the unfixed sample. To prepare fixed cells for FISH, five replicates of 2 ml bacterial culture were centrifuged for 5 min at 8000 × g at 4°C. The resulting cell pellets were resuspended in 1% (v/v) formaldehyde solution (prepared in 1× PBS; 0.2 μm filter sterilized) for fixation overnight at 4°C. Cells were centrifuged again at 8000 × g for 5 min at 4°C. The cell pellets were washed with 1× PBS and resuspended in 1:1 of 1× PBS and 96% (v/v) ethanol (0.2 μm filter sterilized) resulting in fixed samples. To enumerate fixed cells, cell suspensions were filtered onto polycarbonate 0.2 μm pore size filters, mounted in embedding medium consisting of a 3:1 solution of Citifluor/Vectashield and 1 ng/μl of 4′,6-diamidino-2-phenylindole. The resulting filter was counted under an epifluorescence microscope and a counting grid. Five sample replicates of 1 × 10^6^ and 5 × 10^5^ fixed cells were collected on individual membranes of a MultiScreen Filter Plate (Millipore Sigma) using 200 mbar of vacuum pressure. Membranes were excised using ethanol-cleaned scalpels and forceps and stored in 1.7 ml microtubes at −80°C.

### Environmental sample collection

Seawater was sampled from March to May 2020 at the long-term ecological research site ‘Kabeltonne’ (54° 11.3′ N, 7° 54.0 E) off the coast of Helgoland (southern North Sea) at 1 m depth as described previously [23, 28]. For metaproteomics, 10 l of seawater were sequentially filtered using polycarbonate filters (142 mm diameter) of pore-size 10, 3, and 0.2 μm. Filters were flash frozen in liquid nitrogen and stored at −80°C. The 0.2 μm filters containing the bulk of free-living bacteria were used for further preparation. For the FISH-FACS proteomics workflow, seawater was fixed with 37% (v/v) formaldehyde (final concentration 1% (v/v)) for 1 h at room temperature. For each sampling date, 500 ml fixed seawater was sequentially filtered using vacuum pumps at a maximum pressure of 200 mbar through 10, 3, and 0.2 μm pore-size polycarbonate filters (47 mm diameter) to size fractionate the samples and to avoid clogging the filter. Filters were stored at −80°C.

### Fluorescence *in situ* hybridization

Hybridization of formaldehyde-fixed *Polaribacter* sp. KT25b cells from low biomass samples and bacterial biomass cells from environmental samples (on filters) were carried out based on a previously described FISH protocol using tetra-labelled Atto-488 fluorescent probes (Biomers) [29]. The oligonucleotide probe CF319a, targeting most marine *Flavobacteria*, was used to hybridize fixed *Polaribacter* sp. KT25b cells. For environmental samples, *Aurantivirga*-specific probe AUR452 with competitors (AUR452-c1 and AUR452-c2) were used for the target population. Negative controls for FACS were performed using cells hybridized with probe NON338. Probes used in this study are described in Supplementary Table S1. For hybridization of *Polaribacter* sp. KT25b cells in liquid, formaldehyde-fixed cells were centrifuged at 8000 × g at 4°C for 5 min and resuspended in 100 μl of 1× PBS. 15 μl of cell suspension was added to 300 μl of hybridization buffer (900 mM NaCl, 20 mM Tris-HCl pH 8.0, 0.01% (w/v) SDS) containing 35% (v/v) formamide and 3.7 nM probe. The cells were hybridized at 48°C for 3 h, followed by centrifugation and resuspension in prewarmed hybridization buffer without probe at 46°C for 15 min. Subsequently, samples were centrifuged at 8000 × g at 40°C for 2 min and resuspended in ice-cold 1× PBS. Fluorescence microscopy was used to count the FISH-labelled cells by filtering them onto a polycarbonate 0.2 μm pore-size filter, counting them as previously described for generation of low biomass fixed samples. Based on microscopic counting, five replicate samples containing 1 × 10^6^ and 5 × 10^5^ FISH hybridized cells were collected on a MultiScreen Filter Plate (Millipore Sigma) using 200 mbar of vacuum pressure. Membranes were excised using ethanol-cleaned scalpels and forceps and stored in 1.7 ml microtubes at −80°C.

To hybridize bacterial cells from environmental samples, one-quarter (target probe) or 1/20^th^ (negative control) of each sample filter was treated with a modified hybridization buffer (900 mM NaCl, 20 mM Tris-HCl (pH 8.0), 1% (w/v) blocking reagent, 0.02% (w/v) SDS, 0.1% (w/v) dextran sulphate) containing 20% (v/v) formamide and 8.4 nM probe. The filters were placed on petri dishes in a humid chamber at 46°C for 3 h and washed in 50 ml wash buffer (225 mM NaCl, 20 mM Tris-HCl (pH 8.0), 5 mM EDTA, 0.01% (w/v) SDS) at 48°C. Special precautions were taken to prevent cell loss during handling, such as using petri dishes in a hybridization oven rather than falcon tubes in a water bath. Afterward, the filters were rinsed with ultra-pure water, dried on absorbent paper, and stored at −20°C until FACS.

### Fluorescence-activated cell sorting

Hybridized *Polaribacter* sp. KT25b cells and bacterial cells from environmental samples were sorted on Influx Mariner (BD Biosciences) equipped with four lasers (355 nm, 488 nm, 594 nm, and 640 nm), and filter sets for collecting emission spectra detected on up to 12 photomultiplier tubes (Supplementary Table S2) and small particle forward scatter detector using BD FacsFlow (BD Biosciences) as sheath fluid. The filters containing bacterial cells from environmental samples were cut into ~2 mm^2^ pieces, which were transferred into a microtube containing 1–2 ml ice-cold cell removal buffer (150 mM NaCl with 0.05% (v/v) Tween80) [30]. The suspension was vortexed for 15 min at 4°C. The supernatant was transferred to the Influx sample tubes and run on the sample line with a pressure differential of 0.5–1.0 psi. In order to increase sort yield from limited sample material, filter pieces were kept on ice and resuspended as needed with 100–700 μl 1× PBS using short vortex periods (1–7 min) up to six times. FISH-positive cells (CF319a or AUR452 probes) were excited at 488 nm and flow cytometrically sorted on the basis of forward scatter and green fluorescence illustrated in Supplementary Figs S2–S4. Using a negative control (NON338-probe) autofluorescent events with high yellow and red fluorescence signals were excluded. Sorting was performed on ‘1.5 drop pure’ sort mode to ensure purity and accurate sort counts. The fluorescence microscopy images of *Polaribacter* sp. KT25b cells during FISH-FACS steps are shown in Supplementary Fig. S5. The fluorescence microscopy images of bacterial cells from environmental samples during FISH-FACS steps are shown in Supplementary Fig. S6.

### Proteomic sample preparation of low biomass and sorted environmental samples (direct digestion)

A total of 32 μl ammonium bicarbonate (25 mM, pH 7.8) and 2 μl acetonitrile (ACN, 100%) was added to samples. Formaldehyde fixed cells were heated at 98°C in a heating block for 20 min to counteract the fixation. Cells were disrupted using ultrasonication in a microtube holder with a BR-30 probe (Bandelin, Sonoplus) at 100% amplitude for 2 min. All samples were kept at room temperature for 5 min. Afterwards, proteins were proteolytically digested with 8 μl trypsin solution (1 μg, prepared from a stock of 20 μg in 160 μl HPLC grade water) for 2 h at 37°C at 400 rpm in a Thermomixer (Eppendorf). Afterwards, all samples were centrifuged at 13 000 × g at room temperature and supernatants were collected in new 1.7 ml tubes. The supernatants were dried in a vacuum centrifuge. The dried peptides were resuspended in 0.1% (v/v) acetic acid in HPLC grade water and desalted using ZipTips C18 (Merck Millipore, P10 tip size) according to the manufacturer’s protocol. Peptides were eluted in 10 μl 60% (v/v) ACN, 0.1% (v/v) acetic acid and dried in vacuum centrifuge. Finally, peptides were resuspended in 10 μl 0.1% (v/v) acetic acid in HPLC grade water and stored at −80°C until LC–MS/MS analysis.

### Proteomic sample preparation of high biomass samples

*Polaribacter* sp. KT25b cell pellets from three biological replicates of glucose or laminarin-grown cells, respectively, were resuspended in 50 mM Tris-HCl (pH 8.0). Glass beads (0.1 mm, BioSpec Products) were added to the cell pellets for disruption using Ribolyser (FastPrep-24 5G, MP biomedical) for 30 s and 4 cycles at 6.5 m/s speed. Afterwards, samples were centrifuged at 4°C at 20 000 × g for 30 min. The resulting supernatant was collected in new 1.7 ml tubes and centrifuged again at 4°C at 20 000 × g for 30 min. Protein determination of this supernatant was carried out using Pierce BCA Protein Assay Kit (Thermo Fisher Scientific), according to the manufacturer’s procedure. 50 μg of protein was digested using S-Trap (suspension trap, ProtiFi) according to the manufacturer’s procedure with slight changes. About 2× SDS lysis buffer (10% (w/v) SDS in 100 mM triethylammonium bicarbonate (TEAB), pH 7.5) was added to the samples in a 1:1 ratio. Later, proteins were reduced with 500 mM 1,4-dithiothreitol (DTT) at 95°C for 10 min and alkylated with 500 mM iodoacetamide (IAA) for 30 min in the dark at room temperature. Samples were acidified with 1.2% (v/v) phosphoric acid, diluted to a ratio of 1:7 with S-Trap solution (90% (v/v) methanol in 100 mM TEAB) and vortexed thoroughly. Samples were loaded on the S-Trap microcolumn and washed with S-Trap solution. Afterwards, proteins were digested with 20 μl trypsin (Promega) solution (1 μg, prepared from a stock of 20 μg trypsin in 400 μl 50 mM TEAB) with 1:50 (trypsin:protein) ratio at 47°C for 3 h in a Thermomixer (Eppendorf). Peptides were eluted from the column using three solvents as follows: 50 mM TEAB, 0.1% (v/v) acetic acid, and 60% (v/v) ACN in 0.1% (v/v) acetic acid. Peptide concentration was determined using Pierce Quantitative Fluorometric Peptide Assay (Thermo Fisher Scientific). To reduce the complexity, 6 μg of peptide samples were fractionated using basic reversed peptide fractionation. Briefly, peptides were loaded on columns (Pierce Micro-Spin Columns, Thermo Fisher Scientific) containing C18 material (Dr. Maisch HPLC GmbH ReproSil pur C18-AQ, particle size 5.0 μm, pore size 300 Å, 1.9 μm diameter) and eluted sequentially with increasing concentrations of triethylamine (0.1% (v/v)). The resulting eight fractions were orthogonally pooled into pairs (1 & 5, 2 & 6, 3 & 7, and 4 & 8), vacuum centrifuge dried, and stored at −80°C until LC-MS/MS analysis.

### Metaproteomic sample preparation of unsorted environmental samples

The 0.2 μm free-living bacterial fraction was collected during the spring phytoplankton bloom on 5 May 2020 and 7 May 2020 as previously described [31]. In brief, proteins were extracted from approximately one-eighth of the filter (142 mm polycarbonate membrane filters, Millipore) containing biomass using resuspension buffers (composition as previously described) [31, 32]. Cell lysis was performed using ultra-sonication with intermittent cooling on ice. The supernatants were collected, and proteins were precipitated with precooled trichloroacetic acid to a final concentration of 20% (v/v). The protein pellets were washed with precooled acetone, dried at room temperature, and resuspended in 2× SDS sample loading buffer. The samples were heated, separated on SDS-PAGE (Criterion TG 4-20%, BIO-RAD Laboratories Inc.), and stained with Coomassie. The gel lanes were then cut into 20 pieces. After destaining the gel pieces, they were allowed to dry. Proteins were in gel reduced with DTT and subsequently alkylated with IAA. After washing and drying of the gel pieces, they were covered with trypsin solution and digested overnight. Peptides were eluted in 0.1% (v/v) acetic acid in HPLC grade water and transferred to a new tube. A second elution was performed using 30% (v/v) ACN, and the eluates were pooled. The sample volume was reduced in a vacuum centrifuge and peptides were desalted using ZipTips C18 (Merck Millipore, P10 tip size) following the manufacturer’s protocol. The eluted peptides were resuspended in 0.1% (v/v) acetic acid in HPLC grade water, which included 0.5× iRT standard kit (Biognosys).

### Mass spectrometry

The tryptic peptides from direct digestion of low biomass samples and sorted environmental samples were separated on an Easy nLC-1000 liquid chromatography system (Thermo Fisher Scientific, Waltham, USA) with a reverse-phase C18 column (in-house packed, inner diameter 75 μm, length 200 mm, packed with Dr. Maisch ReproSil pur C18, pore size 120 Å, particle size 1.9 μm). Peptides were loaded with 9 μl of 0.1% (v/v) acetic acid in HPLC-grade water at 500 bar and subsequently eluted with 180 min gradient from 1% to 99% of 0.1% (v/v) acetic acid in ACN. Eluting peptides were measured on a Q Exactive mass spectrometer (Thermo Fisher Scientific, Waltham, USA) in data-independent acquisition (DIA) mode with HCD fragmentation. The MS1 scan was recorded in the orbitrap with a mass window of 150–2000 *m*/*z* (AGC target of 3 × 10^6^) and a resolution of 140 000. Then 23 DIA scans were acquired at a variable isolating window and at a resolution of 35 000. The default charge state was set to 2. The resulting MS/MS spectra were acquired in the orbitrap.

The tryptic peptides from high biomass samples and the unsorted environmental samples were separated on an nLC-1000 liquid chromatography system with a reverse-phase C18 column (material and size as specified above). Peptides were eluted with a 145 min gradient (binary nonlinear, 1%−99% 0.1% (v/v) acetic acid in ACN) and measured on the Q Exactive mass spectrometer in data-dependent acquisition (DDA) mode. The scan window for the MS1 scan was set to 300–1650 *m*/*z*, with an AGC target of 3 × 10^6^ and a resolution of 140 000. The top 15 most abundant precursor ions were fragmented (HCD) and MS/MS spectra were acquired in the orbitrap with a resolution of 17 500 at 200 *m*/*z* and a normalized collision energy of 27%.

### Data analysis

The resulting *.raw files acquired from direct digestion of low biomass *Polaribacter* sp. KT25b samples acquired in data-independent mode were analyzed in Spectronaut (v18.6.231227) using *Polaribacter* sp. KT25b protein database from UniProt (Taxon ID 1855336, 3350 proteins, downloaded on 29 September 2020) [33]. A classic library-based DIA search was performed using the following: precursor *Q*-value cutoff 0.01, precursor PEP cutoff 0.2, protein *Q*-value cutoff (experiment) 0.01, protein *Q*-value cutoff (run) 0.05, and protein PEP cutoff 0.75. The library settings include 0.01 peptide FDR, protein FDR and PSM FDR. The library contained 73 123 precursors accounting for 42 898 proteotypic peptides and 2891 proteins (86% of annotated proteins). The resulting data were filtered for reverse sequences and ≥2 precursors identified. Protein quantification values (maxLFQ) were log-transformed and normalized by subtracting the median for relative label-free quantification (LFQ). Samples were categorically annotated based on the protocol step, and quantification values were filtered using the median as the average for three out of five replicates in Perseus (v2.0.3.0) [34].

The *.raw files acquired from direct digestion of FISH-FACS-sorted environmental samples in data-independent acquisition mode were analyzed in Spectronaut (v18.6.231227). An *Aurantivirga* spp. specific database derived from bacterioplankton metagenomic data (metagenome-assembled genomes [MAGs]) from a research study conducted on the same seawater samples off the coast of Helgoland roads, was used for the analysis [23]. The redundancy of the database was reduced to 97% similarity using CD-Hit (v4.8.1) [35] and finally contained 6462 sequence entries. A spectral library was generated from *.raw files of eight environmental samples (seven DIA and one DDA measurements, all are available in the PRIDE repository (ID: PXD057908) resulting in 4995 precursors accounting for 2238 proteotypic peptides. The resulting data were filtered for reverse sequences and ≥2 precursors identified.

The *.raw files acquired from high biomass samples in data-dependent acquisition mode were analyzed in FragPipe (v20.0) with a backbone of MSFragger (v4.0) [36], IonQuant (v1.10.12) [37], and Philosopher (v5.1.0) [38]. In addition to the same UniProt *Polaribacter* sp. KT25b database used as mentioned above, common contaminants and an equal number of decoy sequences were appended. MSFragger searches were performed with two missed cleavages, a maximum of three variable modifications including oxidation M (+15.9949) on a peptide, and carbamidomethylation C(+57.02146) on cysteine as fixed modification. Validation and 1% FDR filtering were performed in Philosopher for proteins, peptides, and PSMs. Identified PSMs were further quantified in IonQuant with two maxLFQ minimum ions and match-between-runs ion FDR set to 1%. The proteins in the final output file were filtered for contaminants and ≥ 2 combined total peptides. The downstream analysis of above-mentioned datasets was performed in Perseus (v2.0.3.0) [34] and data visualization in R (v4.3.3) [39–41].

The *.raw files acquired from unsorted environmental samples (referred to as metaproteomics samples) were analyzed using Mascot (v2.7.0.1; Matrix Science, London, UK) [42] with a metagenome-derived protein sequence database specific to the sampling days (5 May 2020 and 7 May 2020). Redundant protein sequences entries in the database with more 97% sequence identity were clustered using CD-Hit (v4.8.1), resulting in 413 111 sequence entries [35]. Common laboratory contaminants and reverse sequence entries were added to the database (826 222 entries in total). Database searches were conducted as previously described [31]. In brief, Mascot searches were performed with the following settings: fragment and parent ion mass tolerance of 10 ppm, fixed modification on cysteine (+57.0215, carbamidomethylation), and variable modification on methionine (+15.9949, oxidation). Scaffold (v5.3.3, Proteome Software Inc., Portland, USA) [43] was used to validate MS/MS-based peptide and protein identifications. Additionally, X! Tandem Alanine (v2017.2.1.4, The GPM, thegpm.org) was used for validation with default settings [44]. Peptide identifications were set to a threshold of 95%, and protein identifications to a threshold of 99% with a requirement of at least two identified peptides. Proteins with similar peptides were grouped to adhere to the principle of parsimony. The taxonomic annotations for the metaproteomics database were created using a nonredundant Helgoland-specific MAG database [23]. Protein sequences were mapped to these MAGs using diamond blastp (v2.1.1.155) [45] with following flags—evalue 1E-4—id 95—query-cover 70—subject-cover 70. Remaining unmapped sequences were classified as ‘prokaryotic’ or ‘nonprokaryotic’ with BASTA (v1.4.1, options: −m 1 −i 0 −l 0 −e 0.001 −p 90) [46] using diamond blastp (options: —evalue 0.001 -k 100) vs. NCBI nonredundant protein database (‘NCBI_nr’; as of 22 February 2023). For ‘nonprokaryotic’ sequences, the NCBI_nr results were used for Last Common Ancestor (LCA) predictions via BASTA, whereas ‘prokaryotic’ sequences were classified using diamond blastp results against the Genome Taxonomy Database (GTDB; r214.1) with identical thresholds. The functional annotations for MAGs derived database used for sorted environmental samples were performed as follows: Protein sequences were annotated with Bakta (v.1.9.2 [47], invoking Pyrodigal v3.3.0 [48, 49], Pyhmmer v0.10.6 [50, 51], and Diamond v2.1.8 [45]) using default settings and ‘full’ database (release 19 January 2024) as reference. COG (Cluster of Orthologous Groups of proteins) numbers were assigned with reCOGnizer (v1.10.1, invoking RPS-BLAST v2.13.0+) [52] using a maximum *E*-value of 0.01. PUL-relevant protein functions were predicted with hmmscan (v3.3.2) [51] using PFAM [53] models PF07980.15, PF12741.11, PF12771.11, and PF14322.10 for SusD-like proteins and TIGRFAM [54] models TIGR01352, TIGR01776, TIGR01778, TIGR01779, TIGR01782, TIGR01783, TIGR01785, and TIGR01786 (for ‘other’ TonB-dependent receptors) as well as TIGR04056 for SusC-like proteins. CAZymes were predicted using hmmscan vs. dbCAN-HMMdb-V12 [55] and dbCAN-sub [56] as well as diamond blastp (v2.1.1.155, option settings --evalue 1E-20 --id 30 --query-cover 40) against CAZyDB.07262023 [57], provided by dbCAN. All hmmscan results were further parsed using the dbCAN hmmscan-parser.sh script. CAZyme families predicted by at least two tools were considered as correctly assigned.

## Results

**Figure 1:**
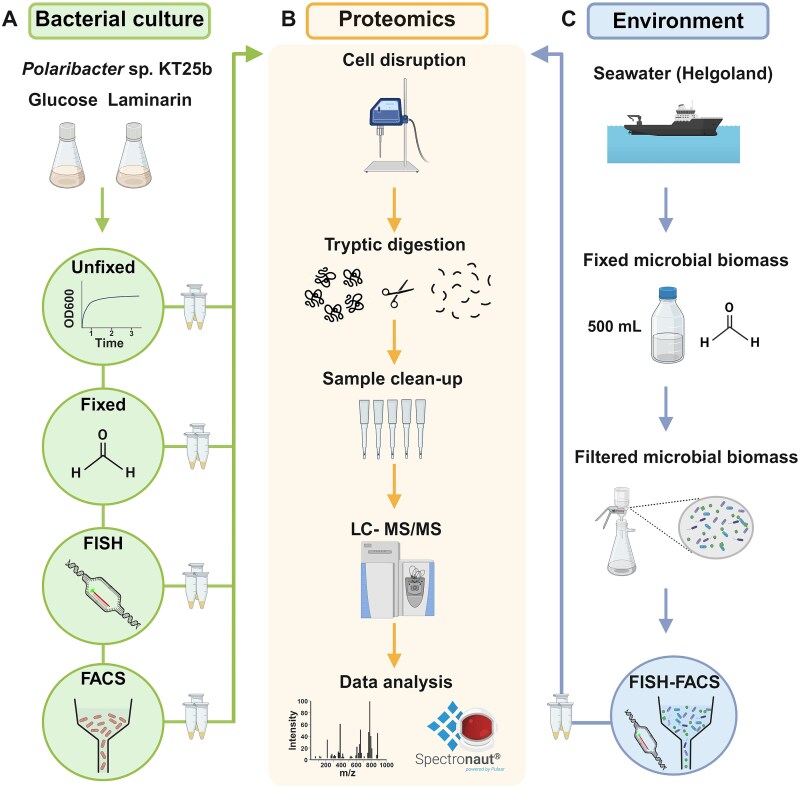
Graphical representation of (A) bacterial culture experiments using *Polaribacter* sp. KT25b to investigate the effects of four sequential steps in the proposed FISH-FACS proteomics workflow on protein identifications. Cells were grown in MPM medium (using either glucose or laminarin as the sole carbon source) and collected at mid-exponential phase (unfixed), followed by formaldehyde fixation, FISH and FACS. To mimic realistic cell numbers collected later from the environment, 1 × 10^5^, 5 × 10^5^, and 1 × 10^6^ cells were collected from the culture for further analysis. (B) Proteomics sample preparation (direct digestion) workflow for FISH-FACS samples. (C) FISH-FACS workflow applied to formaldehyde fixed and filtered sea water samples collected from Helgoland (southern North Sea). The highlighted circles in (A) and (C), respectively, indicate the sample collection points processed through the proteomics preparation, as depicted by microtubes. Created in BioRender: https://BioRender.com/s26f787.

This study aimed to establish a workflow for analyzing proteins from uncultured microorganisms from complex environmental samples. As test organisms, we targeted the flavobacterial sister genera *Polaribacter* and *Aurantivirga*. Both occur in high numbers in marine water samples from the North Sea during spring phytoplankton blooms [23], but only *Polaribacter* could by isolated and cultured so far. To target this bacterial genus, we combined FISH and FACS with MS-based proteomics. The steps in the proposed workflow, namely formaldehyde fixation, FISH, and FACS, were evaluated by the number of proteins identified using cultured and unfixed *Polaribacter* sp. KT25b cells as a reference (Fig. 1A). The challenges of protein identification from low biomass samples were overcome using a straightforward one-pot sample preparation approach (Fig. 1B). To assess the ability of the proposed FISH-FACS proteomics workflow for identification and quantification of proteins from low number of bacterial cells, it was benchmarked against conventional high biomass proteomic sample preparation. The examples of mass spectra acquired from *Polaribacter* sp. KT25b samples and high biomass sample are presented in Supplementary Fig. S7.

We present the first application of this workflow to environmental samples collected off the coast of Helgoland Roads (Fig. 1C).

### Fluorescence microscopy of flow sorted target cells

In order to check the cell integrity and FISH signals based on fluorescence microscopy, cells were examined under the microscope to make qualitative assessments on signal strength and cell appearance. Microscopy and flow cytometry confirmed successful staining of cells in *Polaribacter* sp. KT25b (Supplementary Figs S2 and S5) and in environmental sorted cells (Supplementary Figs S3, S4, and S6). A noticeable decrease in FISH signal intensity after resuspension of cells with vortex action, and after cell sorting was also observed. This is most likely due to some detachment of fluorescently-labelled probe from the rRNA target.

### Protein identifications from FISH-FACS proteomics samples

**Figure 2:**
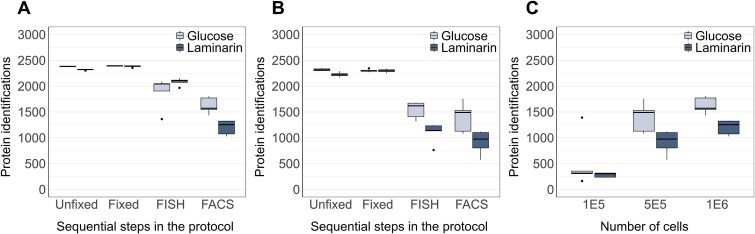
The effect of sequential steps in the workflow on the number of protein identifications. The strain *Polaribacter* sp. KT25b was cultivated in MPM medium using either glucose or laminarin as the sole carbon source. Cells were collected at different stages during the workflow, named unfixed, fixed, FISH, and FACS (Fig. 1A). (A) Protein identifications (median value highlighted in bold horizontal line) from 1 × 10^6^ cells; (B) 5 × 10^5^ cells. (C) The effect of the number of sorted cells on protein identifications yielded by the FISH-FACS proteomics workflow.

The optimization of the FISH-FACS proteomics workflow was hampered by the limited availability of suitable biomass from the environment. Therefore, we used defined cell numbers of the marine model bacterium *Polaribacter* sp. KT25b to mimic the available environmental biomass and protein amount. An estimation of the environmental availability of target bacterial cell number was inferred from the microscopic counts using fluorescently labelled catalysed reporter deposition-FISH probes (e.g. AUR452 for *Aurantivirga*) described in the study of the spring bloom in the German Bight in 2020 [23]. To evaluate whether quantitative protein information can be preserved throughout the extensive sorting workflow, well-established and easily accessible experimental conditions were selected for comparison [20]. Therefore, we cultivated *Polaribacter* sp. KT25b with two carbon sources: glucose and laminarin. Cells were collected during mid-exponential phase (Supplementary Fig. S1). Throughout the workflow, cells were sequentially collected to track the effect on protein identifications (Fig. 1A). We collected 1 × 10^6^ and 5 × 10^5^  *Polaribacter* sp. KT25b cells in five replicates to ensure robust data on the reproducibility of our downstream analyzes. The defined number of cells (specifically 1 × 10^6^) resulted in ~100 ng of starting protein amount (Supplementary Fig. S8). LC–MS/MS analysis revealed reproducible protein identifications across all samples prepared with the direct digestion method (Supplementary Table S3).Cells collected in the unfixed state yielded on average 2384 protein identifications in five out of five replicates from 1 × 10^6^ cells grown in glucose and 2319 from laminarin samples (Fig. 2A). The application of the direct digestion method to these samples resulted in identification and quantification of ~71% of the total annotated proteins of this bacterium. Fixation with 1% formaldehyde before FISH did not decrease the number of protein identifications. Following FISH, protein identifications in glucose-grown cells dropped by 20% on average, down to 1888 proteins. After FACS, the number of identifiable proteins decreased further, with a 32% reduction, averaging 1625 proteins. For cells grown in laminarin, a 10% decline in protein identifications following FISH (average: 2084) and 50% post-FACS (average: 1202) was noted. For 5 × 10^5^ unfixed cells, we observed comparable protein identifications, with an average of 2319 for glucose-grown samples and 2228 for laminarin-grown samples (Fig. 2B). The effect on protein loss during intermediate steps in the protocol was more prominently observed in 5 × 10^5^ cell samples, specifically after FISH and FACS.

To check whether any step in the proposed workflow introduces bias in the protein composition of the sample, identified proteins were examined using the *in silico* prediction tool PSORTb (v3.0.3) [58]. Prediction of subcellular localization indicated only minor changes in the distribution of the cellular localization of proteins identified throughout the workflow. For both 1 × 10^6^ and 5 × 10^5^ cells, the distribution remained comparable to the predicted proteome from the UniProt database (Supplementary Fig. S9A and B). To demonstrate the reproducibility of direct digestion in post-FACS cells, along with 5 × 10^5^, and 1 × 10^6^, we collected as low as 1 × 10^5^ cells. As expected, we observed a direct proportionality between the number of cells and the number of protein identifications (Fig. 2C). On average around 300 proteins could be identified from 1 × 10^5^ cells, which represents only one-tenth of the available cell number of this target bacterial clade in our representative environmental samples.

### Evaluation of the decline in protein identifications

**Figure 3:**
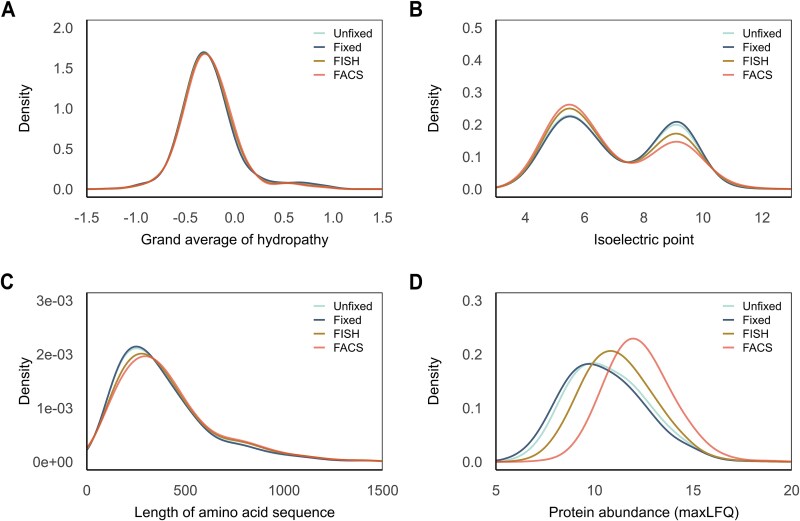
Physicochemical properties of proteins identified in five out of five sample replicates in a density plot of 1 × 10^6^  *Polaribacter* sp. KT25b cells grown in either glucose or laminarin as carbon source. Individual lines represent the sequential steps in the protocol. The distribution of identified proteins based on (A) the grand average of hydropathy, (B) the isoelectric point, (C) the length of the amino acid sequence, and (D) protein abundance represented with maxLFQ values.

To further investigate the decline in protein identifications observed during the sequential steps in the workflow, we analyzed the proteins based on their physicochemical properties, using the unfixed cells as reference. Overall, the distribution of all protein properties overlapped after fixation with formaldehyde (Fig. 3). The distribution of both hydrophilic and hydrophobic proteins identified remained consistent throughout the workflow (Fig. 3A). At the same time, we noted an increase in acidic proteins and a decrease in basic proteins after using the FISH and FACS techniques (Fig. 3B). Furthermore, depending on the amino acid sequence length, we observed minor loss in small proteins after cell sorting (Fig. 3C). While the analysis of physical properties exhibited only minor differences in the proteins identified throughout the workflow, the analyzes of protein abundance showed a significant loss of low abundant proteins after FISH and FACS (Fig. 3D). However, after median normalization of quantitative values of identified proteins, the distribution was similar and thus comparable throughout the workflow. Similar results were obtained from 5 × 10^5^ cells (Supplementary Fig. S10).

### Impact of FISH and FACS on protein quantification

**Figure 4:**
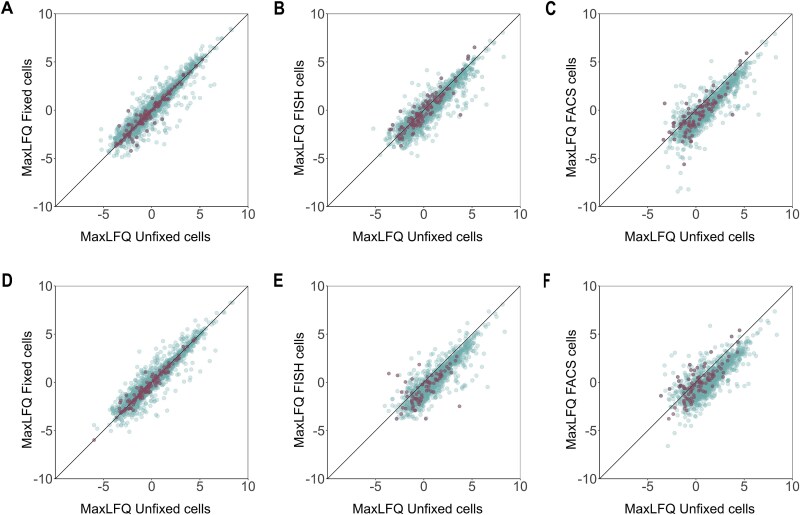
Correlation between quantification values (maxLFQ) of identified proteins. Data represents proteins quantified from 1 × 10^6^  *Polaribacter* sp. KT25b cells (A, B, and C) and 5 × 10^5^ cells (D, E, and F) grown with glucose. Highlighted proteins are carbohydrate-active enzymes and TonB-dependent transporters.

To check whether quantitative information on protein abundance is retained throughout the workflow, maxLFQ values for quantifiable proteins were compared at each step, using the unfixed state of the cells as reference. A positive Pearson correlation was observed for samples stemming from 1 × 10^6^ and 5 × 10^5^ unfixed cells compared to fixed, FISH, and post-FACS cells grown in glucose (Fig. 4). This was also observed in laminarin grown cells (Supplementary Fig. S11).

Of note, an effect on protein quantification post-FACS was more pronounced after scaling down the number of cells as depicted by scattering of quantification values (maxLFQ) in Fig. 4C and F. However, proteins of interest for the current study, specifically the CAZymes and TBDTs (Fig. 4 [highlighted]) retained quantification values after cell sorting.

### Analysis of differential protein abundance

**Figure 5:**
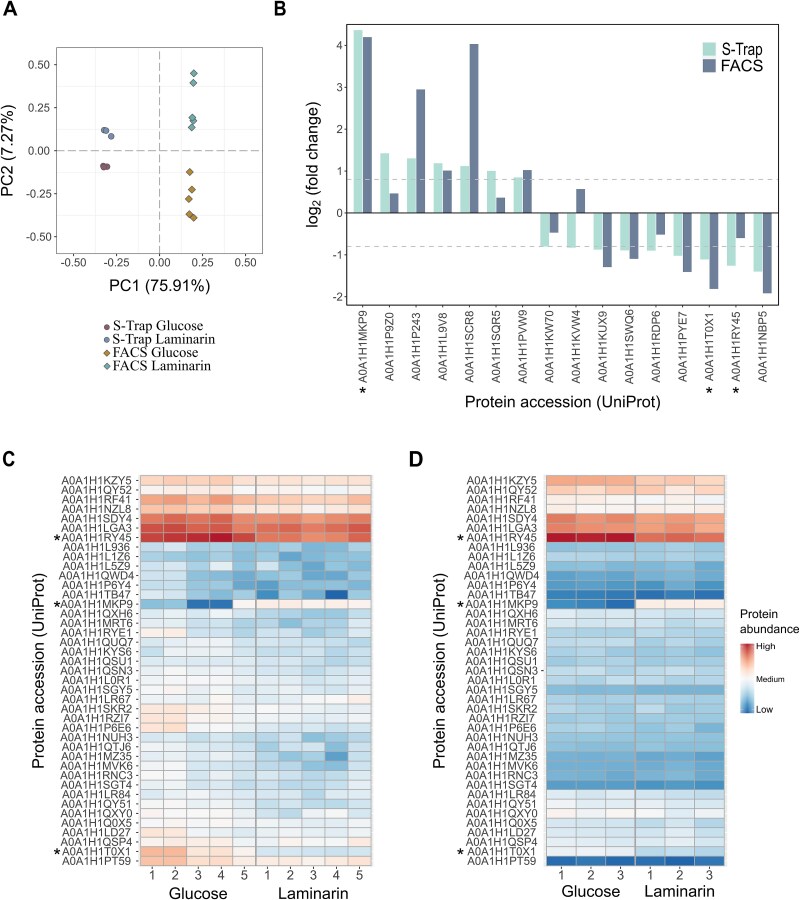
Protein abundance analysis based on label-free quantification (maxLFQ) values. (A) PCA plot of proteins commonly identified in samples prepared from S-trap (unsorted high biomass samples) and FISH-FACS proteomics (1 × 10^6^ cells) workflow. (B) Comparison of fold change of differentially abundant proteins (FC (>|0.8|) in samples prepared from S-trap and FISH-FACS proteomics (1 × 10^6^ cells) workflow. (C, D) Heatmap visualization of protein abundance of carbohydrate-active enzymes and TonB-dependent transporter in samples prepared (C) from 1 × 10^6^ sorted *Polaribacter* sp. KT25B cells and (D) via S-trap (unsorted high biomass samples). * Proteins of interest discussed in this study.

To compare the quantitative protein data from the FISH-FACS proteomics workflow with that from unsorted samples with nonlimited protein amounts (high biomass samples), 50 μg of protein from unfixed cells was digested using the widely applied S-Trap protocol [59]. Since the S-Trap method is well-established and protein quantity was not a limiting factor in these experiments, three replicates of the samples were analyzed. Subsequently, a principal component analysis (PCA) of label-free quantification (maxLFQ) values was conducted on high biomass samples and 1 × 10^6^ sorted cell samples (Fig. 5A). PCA showed high reproducibility within sample replicates from both methods and a separation between cells grown in glucose or laminarin. Furthermore, proteins quantified as differentially abundant depending on the carbon source (*P*-value <.05) in both high biomass samples and sorted samples exhibited a consistent abundance pattern with acceptable deviations (Fig. 5B). Ten out of 16 proteins, including exo-beta-1,3-glucanase (GH17 family, A0A1H1MKP9), alanine dehydrogenase (A0A1H1P243), glycogen recognition site of AMP-activated protein kinase (A0A1H1L9V8), fructose-1,6-biphosphatase class 1 (A0A1H1KUX9), and outer membrane receptor proteins mostly involved in Fe transport (A0A1H1T0X1, A0A1H1NBP5), exhibited the same abundance in both datasets. Five proteins, namely glyceraldehyde-3-phosphate dehydrogenase (A0A1H1P9Z0), iron complex outer membrane receptor protein (A0A1H1RY45), superoxide dismutase (A0A1H1KW70), peptidyl-prolyl cis-trans isomerase (A0A1H1SQR5), and Por secretion system C-terminal sorting domain-containing protein (A0A1H1RDP6), showed similar abundance patterns but did not meet the fold change (FC) cut-off criteria to be considered as biologically relevant (>|0.8|) for sorted samples. Only cobalt-zinc-cadmium resistance protein CzcA (A0A1H1KVW4) exhibited contrary fold changes, indicating poor quantification within sample replicates in both datasets. Since proteins involved in polysaccharide utilization are a focus of our proof-of-concept study, their abundance was examined in greater detail across sample replicates in both datasets. A total of 41 quantified proteins of interest were visualized in a heatmap generated from data derived through the FISH-FACS proteomics workflow or S-Trap method (median normalization performed individually for both datasets) (Fig. 5C and D). A similar protein abundance trend within sample replicates was observed in both datasets. Notably, exo-beta-1,3-glucanase (GH17 family, A0A1H1MKP9), was upregulated in laminarin-grown cells compared to glucose-grown cells (FC (S-Trap) = 4.36; FC (FISH-FACS proteomics) = 4.19) and is thus also included in Fig. 5B. It is a glycoside hydrolase listed in the carbohydrate-active enzymes (CAZy) database and is involved in depolymerization and degradation of high molecular weight compounds such as laminarin [60]. These results resemble *in vitro* and *in situ* expression data of the laminarin utilizing marine bacterium *Formosa* sp. Hel1_33_131 [61].

Two membrane proteins involved in the facilitated diffusion of large molecules, such as oligosaccharides and ferric chelates, across the cytoplasmic membrane [24], were downregulated in laminarin-grown cells compared to glucose-grown cells in both datasets (A0A1H1T0X1: FC (S-Trap) = −1.10, FC (FISH-FACS proteomics) = −1.81); A0A1H1RY45: (FC (S-Trap) = 1.26, FC (FISH-FACS proteomics) = 0.6). The detection of differentially abundant proteins of *Polaribacter* sp. KT25b, grown with altered carbon sources, was successfully retained in the FISH-FACS proteomics samples, highlighting its potential for application to environmental samples. The differential protein abundance analysis results for the 5 × 10^5^ sorted cell samples were consistent with those obtained from the 1 × 10^6^ cells (Fig. 5, Supplementary Fig. S12). However, in these samples, the degree of missing values increased due to low peptide load, which caused the peptide amount to fall below the detection threshold of the MS.

### Application of the FISH-FACS proteomics workflow to environmental samples

Surface seawater samples collected from Kabeltonne Helgoland in 2020 were used to evaluate the applicability of the FISH-FACS proteomics workflow on environmental samples. The key genus *Aurantivirga* was targeted due to its high abundance during the North Sea algal bloom in 2020 [23]. As no reference genome has been sequenced from an *Aurantivirga* spp. isolate, a protein database was created using bacterioplankton MAGs from the same seawater samples. A total of 1.5 × 10^6^ and 1.2 × 10^6^ cells were sorted on 4 May 2020 and 8 May 2020, respectively. The gating strategy for FACS of the environmental sample from 8 May 2020 is shown as a representative example in Supplementary Figs S3 and S4. The gated population comprised between 6% and 9% of the total which was sorted at an average sort rate between 75 and 154 sorts per second with >94% efficiency. A library-based identification of mass spectra using Spectronaut revealed 3813 *Aurantivirga* spp. peptides for the samples from 4 May 2020 to 8 May 2020 (Supplementary Table S4). Out of the peptides identified on these two sampling days, 3390 peptides were commonly identified on both days, while 58 peptides were only identified in the samples from 4 May and 365 peptides only from 8 May. Among the proteins mapped to these peptides, several CAZymes were identified and quantified on both days, including endo-1,3-beta-glucanase (GH16), glycosyl hydrolase (GH74), alpha-amylase (GH13), and cellobiose phosphorylase (GH149). Endo-1,3-beta-glucanase is known to hydrolyse the glycosidic bonds in β-glucans, such as laminarin, which was found during the North Sea algal bloom [23, 62]. In addition to these, TonB-dependent receptor and SusC/RagA family proteins, which are involved in polysaccharide utilization, were also identified. Ribosomal proteins made up 12% of the identified peptides, with 20 out of 21 annotated proteins for the small subunit and 28 out of 33 large subunit proteins were identified. In a comparable metaproteomics dataset from unsorted cells, analyzed with an MAGs-derived database of 5 May 2020 and 7 May 2020, we identified 65 227 peptides across both days (Supplementary Table S5). However, due to the presence of conserved protein sequences across multiple bacterial clades present in these samples, only 33 peptides were uniquely assigned to *Aurantivirga* spp., while 10 132 peptides assigned to *Aurantivirga* spp. but shared with broader taxonomic levels were identified. These results underscore the need and robustness of the FISH-FACS proteomics workflow for identifying and mapping clade-specific peptides from complex environments.

## Discussion

To understand interactions, functional roles, and environmental adaptability of key microbial clades in a community, we need detailed protein-level insights into nonculturable or yet to be cultured bacteria within microbiomes. Our developed pipeline combines FISH-FACS using 16S rRNA based identification [12], and MS-based proteomics, enabling in-depth study of targeted species. The first challenge in the tested FISH-FACS proteomics workflow was the requirement for cell fixation for FISH labelling. While formaldehyde fixation preserves cell integrity and supports the long-term storage of environmental samples, it has been shown to be incompatible with single-cell genomics [63]. A reasonable assumption would be that formaldehyde fixation may reduce the number of identifiable proteins. Despite this, we found that using 1% (v/v) formaldehyde was compatible with downstream proteomics analysis, with no significant protein loss observed (Figs 2A and B and 3). Based on the literature, we assume that the effect of formaldehyde-induced protein cross-linking was compensated by heating the fixed samples at high temperature [64–67]. This finally led to the identification of 71% of the total annotated proteins for *Polaribacter* sp. KT25b. However, besides fixation, FISH requires specific temperature (46°C) to allow the probe to enter the cell and bind to the intracellular target sequence [68]. The permeabilization of the cell membrane may have caused the loss of small, and low abundant proteins after hybridization (Fig. 3B−D). Nevertheless, 56% of the total annotated proteins were identified in the FISH-FACS proteomics samples from 1 × 10^6^ cells (Fig. 2A and B). Interestingly, among the proteins lost after FISH-FACS, 26% were uncharacterized proteins, while the proteins that were still identified contained only 12% of uncharacterized proteins. Other proteins lost during FISH-FACS include methylmalonyl-CoA epimerase (Uniprot ID: A0A1H1N5Q1), peptidyl-prolyl cis-trans isomerase (A0A1H1LCU9), homospermidine synthase (A0A1H1NZ17). Additionally, proteins involved in carbohydrate metabolism were also lost, such as the SusD family protein (A0A1H1SLU9), outer membrane transport energization protein TonB (A0A1H1SJJ9), and several arylsulfatases.

The second challenge in the FISH-FACS proteomics workflow was to determine the appropriate number of cells to sort which simultaneously provides sufficient starting biomass (protein amount) while maintaining high (>90%) purity of the target population. Until recently, FACS in combination with MS-based proteomics has typically been limited to *in vitro* culturable bacteria and eukaryotes [16, 17, 69–71]. The protein amount in bacterial cells is orders of magnitude less than in mammalian cells. Consequently, while single-cell proteomics by MS (SCP-MS) is a growing field for mammalian cell studies, working with low biomass samples remains challenging for bacterial cells [72, 73]. The protein amount of a bacterial cell ranges in femtograms, and may depend on the cell size and volume [74]. Therefore, bacterial cells have been sorted in a range of 1–6 million resulting in 100−500 ng (Supplementary Fig. S8) of protein amount as a starting material for MS-based proteomics [16, 17, 75]. In our study, we sorted 1 × 10^5^, 5 × 10^5^, and 1 × 10^6^ cells of a cultured isolate to test the lower limit of the proteomics workflow, and determined that 1 × 10^6^ cells are sufficient to derive physiological inferences (Fig. 2).

A method optimization required minimizing the number of sorted cells for two main reasons. First, there are practical constraints on the number of cells that can be sorted in a working day. At the conditions used in this study, the sorting speed is ~60–100 sorts s^−1^ (150 sorts s^−1^ maximum), with an efficiency >90%. This allows for sorting 1 × 10^6^ cells from environmental samples in ~3 h, plus a 2-h start-up time for the FACS instrument. Additional time was required for microscopy checks, handling instrument drift, and performance maintenance. Ultimately, determining the lower limit of input cells was essential to maintain the practicality and feasibility of the experiments. The second limitation on sorted events was due to the restricted sample material collected during the 2020 sampling campaign. To put it into perspective, a 500 ml seawater filtered FISH-FACS sample with a target population of ~5% relative abundance with a total cell count of 1 × 10^6^ cells per ml would yield a maximum of 2.5 × 10^7^ target cells. In practice, loss of target cells occurs at several stages of the sample collection and FISH-FACS protocol, including seawater size-fractionation, cell resuspension from membrane filters [76] and cell sorting. Loss during cell sorting occurs due to exclusion of target cells attached to non-target cells or that exhibit weak FISH signals in order to maintain sort purity.

The third challenge in the FISH-FACS proteomics workflow involved the proteomic sample preparation, including protein extraction, protein digestion and sample clean-up. It is well-known that the different steps in proteomics sample preparation can result in protein and peptide loss, especially when removing incompatible chemicals like detergents, nucleic acids, and chaotropes [77]. However, as the number of sorted bacterial cells from environmental samples is limited, it is crucial to minimize protein loss during sample preparation. We optimized a straightforward one pot in-solution digestion (direct digestion) protocol to get reliable and reproducible protein identification and quantification. Unlike the conventional proteomics experiment, reduction and alkylation of proteins before digestion was omitted in direct digestion protocol. In our preliminary experiments (Supplementary Fig. S13), we observed an increase in the proteins identifications without using these steps. We speculate that the excess amount of trypsin used relative to the low amount of protein sample, likely resulted in sufficient number of tryptic peptides for MS analysis. Additionally, to evaluate the effect of omitting reduction and alkylation steps on identification of cysteine containing peptides we performed *in silico* digestion of all annotated *Polaribacter sp*. KT25b proteins. The analysis revealed that only 10% of peptides contained cysteine residues, and just two proteins required cysteine containing peptides for reliable identification.

Although our experiments focused on a genus-constrained target, the workflow is adaptable to other suitable targets from environmental samples. We consider a suitable target as one that is abundant for sorting (>5%), which have genomes (MAGs or single amplified genomes (SAGs), ideally from the same samples) and validated FISH probes. Careful selection of FISH probe is essential, as too broad probes might not capture enough individual population for downstream proteomic sample preparation, thereby negating the benefits of enrichment. The probe coverage should be evaluated against reference 16S rRNA databases and, if available, custom databases specific to the analyzed environment, to ensure adequate coverage. The use of an existing, well-established FISH probe despite incomplete coverage may be acceptable in order to build on existing work, especially when the sequences in question do not appear to be very abundant or recurrent. In our experiments, the used probe AUR452 does not target the type strain *Aurantivirga profunda* but rather covers the most abundant species clusters which regularly reoccur in Helgoland spring blooms (Supplementary Fig. S14). The per-sample yield could be improved further with adapted protocols during the FISH FACS workflow that minimize cell clumping. Currently, seawater cells are fixed, filtered, and stored on dried filters, but resuspension often results in large clumps containing non-target cells or auto fluorescent debris. This may be due to an adhesive matrix formed by cell debris and extracellular compounds during drying, which is challenging to dissolve. If an alternative sampling and FISH protocol could be devised which avoids cell clumping, the FISH-FACS protocol has further potential for improved sort yield outcomes and for application on even rarer taxonomic groups of interest.

The advancement of metaproteomics applications in environmental studies has significantly enhanced our understanding of a wide array of bacterial species and their metabolic contributions [3, 8]. However, the proteome coverage of a single microbial clade within a complex microbial community is reduced in metaproteomics due to significant variations in protein abundance across different genera [78]. The complexity of metaproteomics samples presents challenges in assigning peptides at the clade-specific level. The workflow presented here enhances sensitivity and coverage for analyzing target bacterial clades on proteome level in complex environmental samples by enabling the use of clade-specific databases for data analysis.

The FISH-FACS proteomics workflow is robust and adaptable, making it particularly suitable for metaproteomics samples containing recurrent abundant bacterial clades. It is a workflow which could be adapted to other environments and applications. For instance, an in-depth metaproteomic analysis of human gut bacterial species identified certain dominant generalist bacteria that mapped the highest number of spectra during MS-based analysis in patients with chronic diseases [79]. This finding suggests that our workflow can be used to specifically investigate these prevalent gut bacterial genera to study disease pathology and progression. Additionally, the presented approach can be applied to various marine systems to draw physiological conclusions about currently uncultured yet abundant bacterial clades [80–83], provided a matching protein database is available. Ideally, this database should be derived from metagenomes from the same sampling site, as demonstrated in this study, but it can also be created using publicly available MAGs [84] or SAGs, the Genome Taxonomy Database [85] after taxonomy-based filtering. Furthermore, adaptation of the workflow to other taxonomic targets requires all the experimental validation steps typical for a standard FISH experiments, along with additional sample replicates to account for potential variability in specific applications.

Overall, the workflow presented here is a broadly applicable method that enables the direct enrichment of uncultured microbes from their habitat and their subsequent proteome analysis. This closes a significant methodological gap and paves the way for more detailed functional proteome-based physiological analyzes of key organisms in complex environmental microbiomes.

## Supplementary Material

Supplementary_information_ycaf145

Supplementary_Table_03_ycaf145

Supplementary_Table_04_ycaf145

Supplementary_Table_05_ycaf145

Supplementary_Table_06_ycaf145

## Data Availability

All the MS data have been deposited to the ProteomeXchange Consortium via the PRIDE partner repository with the dataset identifier PXD057908 (Token: NhW8GTweV2hD) [86, 87]. The sample overview file to distinguish and identify the different experimental MS *.raw files has been submitted to the PRIDE repository and is also provided as Supplementary Table S6. Metagenome, MAG sequence data are available from the European Nucleotide Archive under accession PRJEB52999 (assembly name 2020-05-05-am_CCS, 2020-05-07-am_CCS). The flow cytometry data are deposited to https://doi.org/10.5281/zenodo.14282098.
