# Supplementary material for: FISH-FACS proteomics: enhanced label-free quantitative proteome analysis from low cell numbers of uncultured environmental microorganisms
Source: ISME Commun. 2025 Aug 23;5(1):ycaf145. doi: 10.1093/ismeco/ycaf145 (PMC12452284; doi:10.1093/ismeco/ycaf145)
Supplement: Supplementary_information_ycaf145 [file supplementary_information_ycaf145.docx]

**FISH-FACS Proteomics: Enhanced label-free quantitative proteome analysis from low cell numbers of uncultured environmental microorganisms.**

Vaikhari Kale^1^, G. Y. Grace Ho^2^, Sandra Maaß^1^, Anke-Trautwein Schult^1^, Daniel Bartosik^3,4^, Thomas Schweder^3,4^, Bernhard M. Fuchs^2^, Dörte Becher^1,4^

1. Institute of Microbiology, University of Greifswald, 17489 Greifswald, Germany.
2. Max Planck Institute for Marine Microbiology, 28359 Bremen, Germany.
3. Pharmaceutical Biotechnology, Institute of Pharmacy, University of Greifswald, 17489 Greifswald, Germany.
4. Institute of Marine Biotechnology e.V., 17498 Greifswald, Germany.

* Corresponding author: Dörte Becher, Prof. Dr. Dörte Becher, Institut für Mikrobiologie, Universität Greifswald, Felix-Hausdorff-Strasse 8, Greifswald, 17489, Germany

Email: dbecher@uni-greifswald.de

E-mail addresses of all authors:

Vaikhari Kale vaikhari.kale@uni-greifswald.de

G. Y. Grace Ho gho@mpi-bremen.de

Sandra Maaß sandra.maass@uni-greifswald.de

Anke Trautwein-Schult anke.trautwein-schult@uni-greifswald.de

Daniel Bartosik daniel.bartosik@uni-greifswald.de

Thomas Schweder schweder@uni-greifswald.de

Bernhard M. Fuchs bfuchs@mpi-bremen.de

Dörte Becher dbecher@uni-greifswald.de

Key words: Algae bloom, North Sea, Bacterioplankton, FISH, FACS, proteome, low biomass.


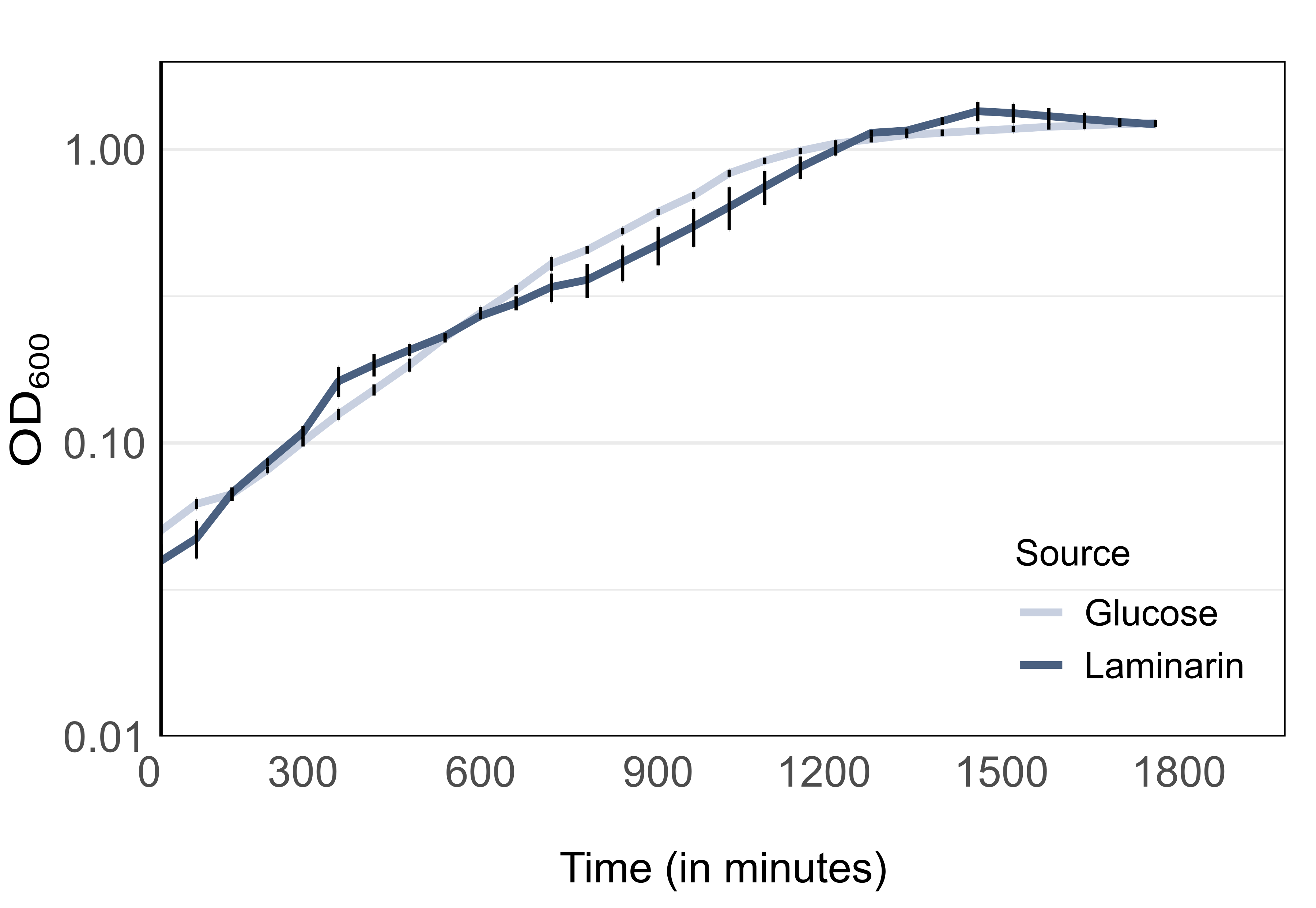


**Supplementary Figure 1:** Growth curve of *Polaribacter* sp. KT25b grown in synthetic seawater medium supplemented with 0.2% (w/v) glucose and laminarin. Cells were harvested after 13 hours (780 min) during the mid-log phase.


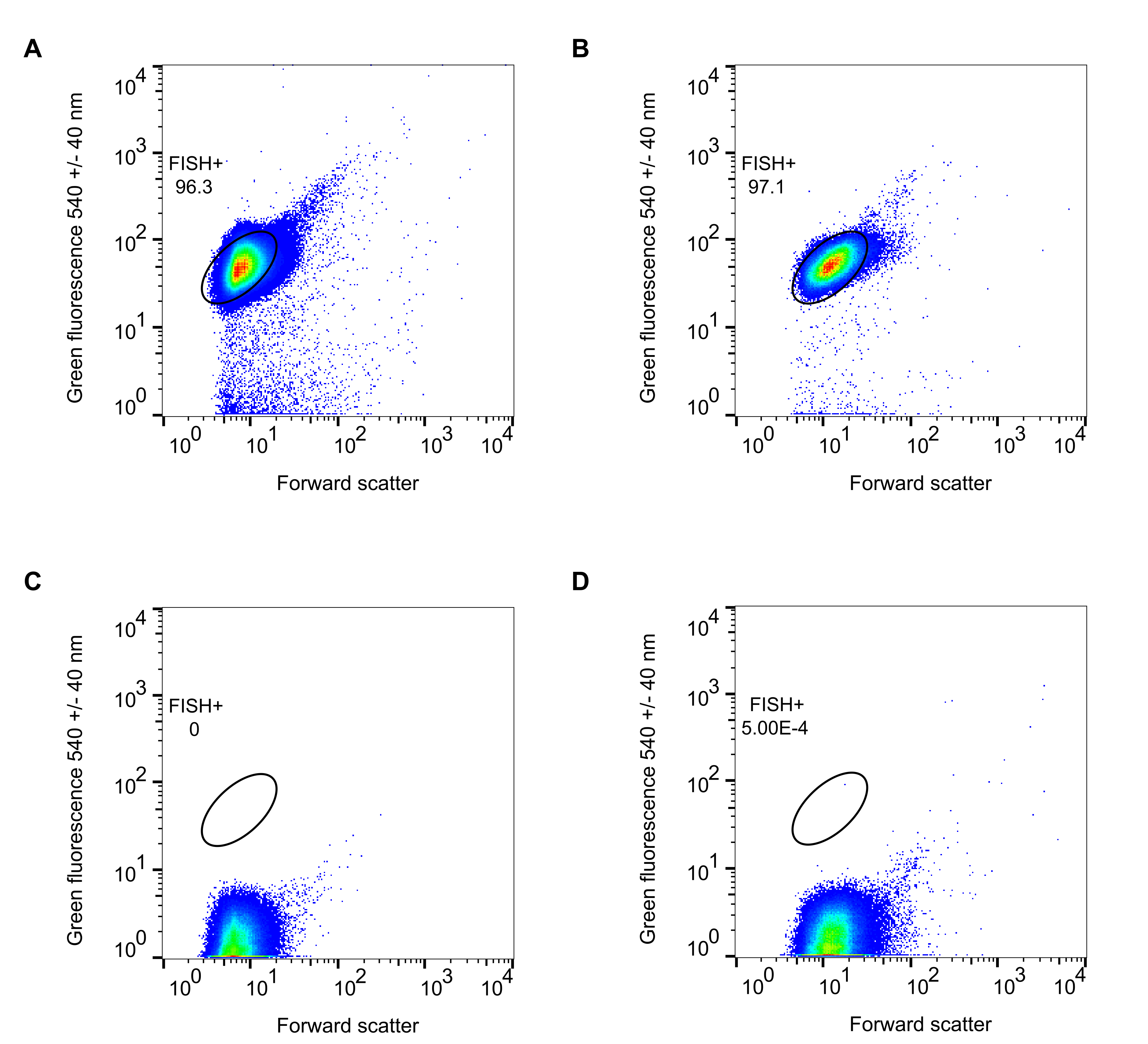


**Supplementary Figure 2:** FACS gating strategy for *Polaribacter* sp. KT25b cells grown in MPM medium supplemented with glucose (**A**, **C**) or laminarin (**B**, **D**), hybridised with CF319a (**A**, **B**) or NON388 probes (**C**, **D**). The gate labelled FISH+ corresponds to the approximate gate drawn in BD FACS Sortware (v.1.2) for sorting, with the number indicating the percentage of total events within the gate. Note that the gate differs between cells grown in glucose and laminarin.


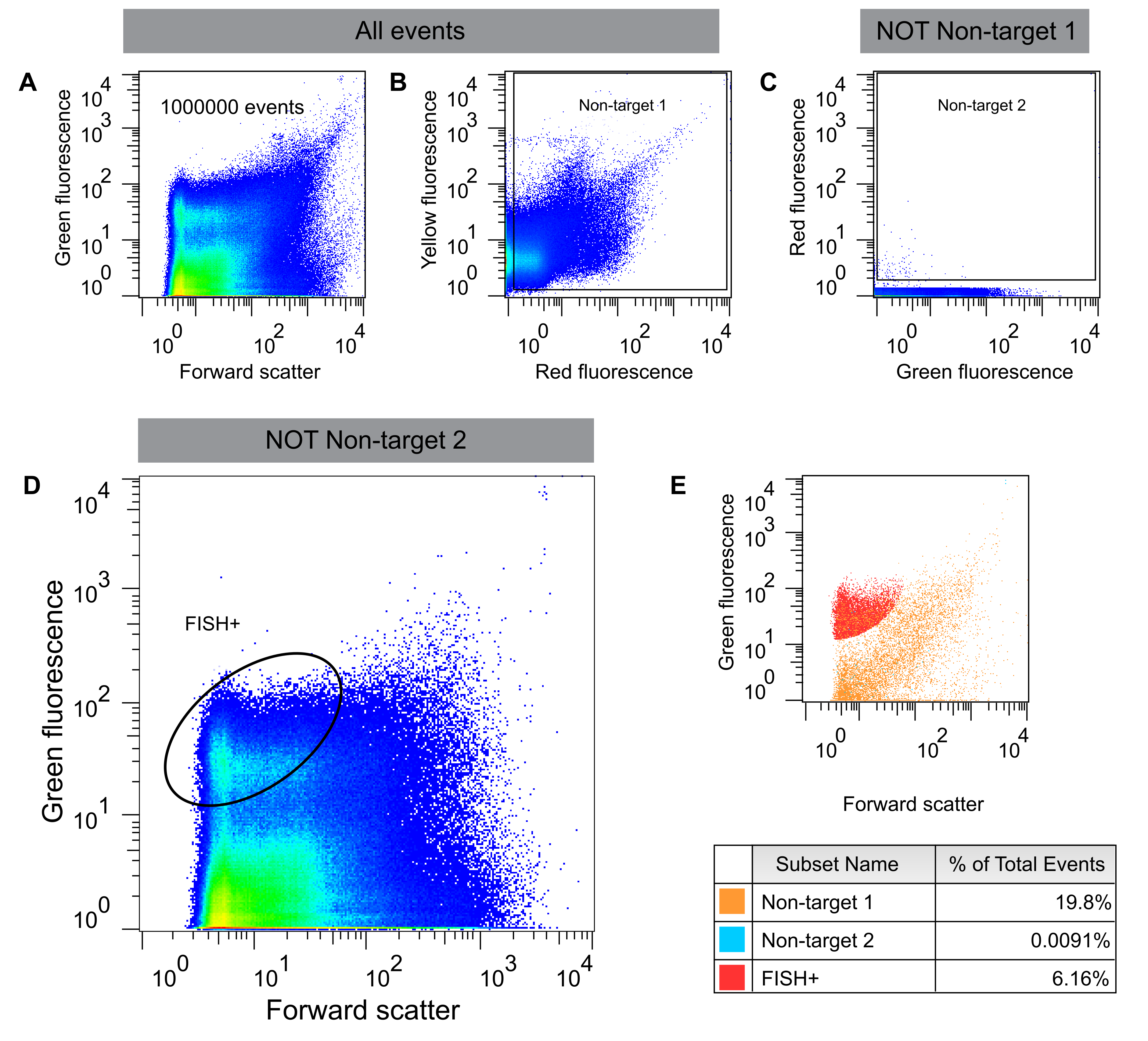


**Supplementary Figure 3:** Flow cytometry data from 1,000,000 events recorded from AUR452-hybridised samples taken on 8 May 2020. (**A**) All ungated events plotted by forward scatter and green fluorescence. (**B**) The exclusion gate “Non-target 1” drawn based on co-occurring red and yellow fluorescence. (**C**) A second exclusion gate “Non-target 2” drawn based on co-occurring green and red signals was drawn to remove any remaining autofluorescent particle. (**D**) Finally, after excluding suspected non-target events, a sorting gate is drawn based on the apparent FISH-positive (“FISH+”) population visible in the green fluorescence channel. (**E**) The different gated cell populations overlaid on the forward scatter-green fluorescence biplot, illustrating the necessity to gate out autofluorescent events. Note that the actual sorting gates were approximated and renamed for reader comprehension in FlowJo (v.10) based on plots exported from BD FACS Sortware (v.1.2). All laser and optical filter parameters are found in **Supplementary Table 2**.


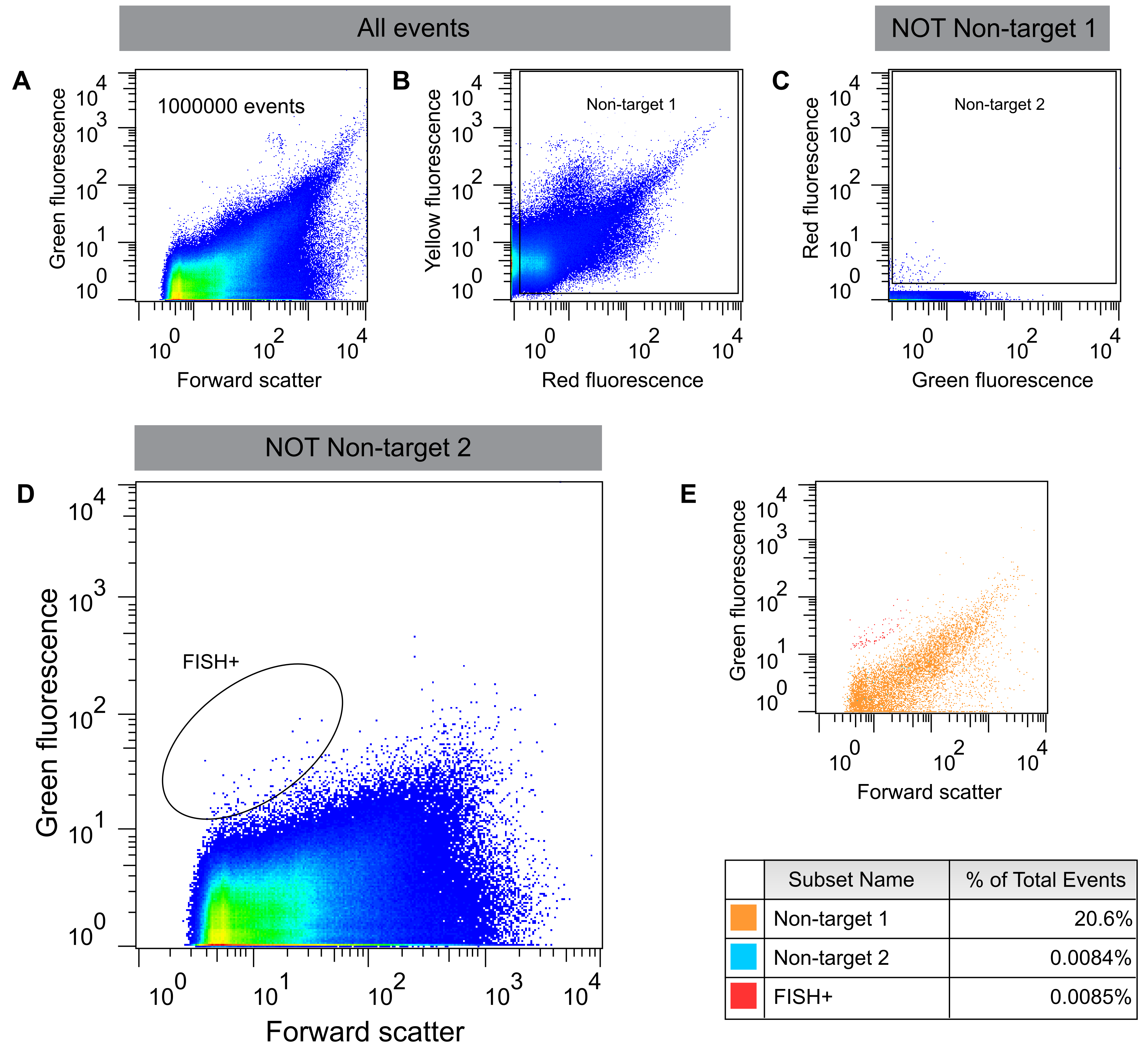


**Supplementary Figure 4:** Flow cytometry data from 1,000,000 events from NON338-hybridised samples collected on 8 May 2020. (**A**) All ungated events plotted by forward scatter and green fluorescence. (**B**) The exclusion gate “Non-target 1” drawn based on co-occurring red and yellow fluorescence. (**C**) A second exclusion gate “Non-target 2” drawn based on co-occurring green and red signals was drawn to remove any remaining autofluorescent particles. (**D**) The sort gate as shown in **Supplementary Fig. 3** (**D**) targeting the FISH-positive population. (**E**) The different gated cell populations overlaid on the forward scatter-green fluorescence biplot, illustrating the necessity to gate out autofluorescent events. Note that the actual sorting gates were approximated and renamed for reader comprehension in FlowJo (v.10) based on plots exported from BD FACS Sortware (v.1.2). All laser and optical filter parameters are found in **Supplementary Table 2**.


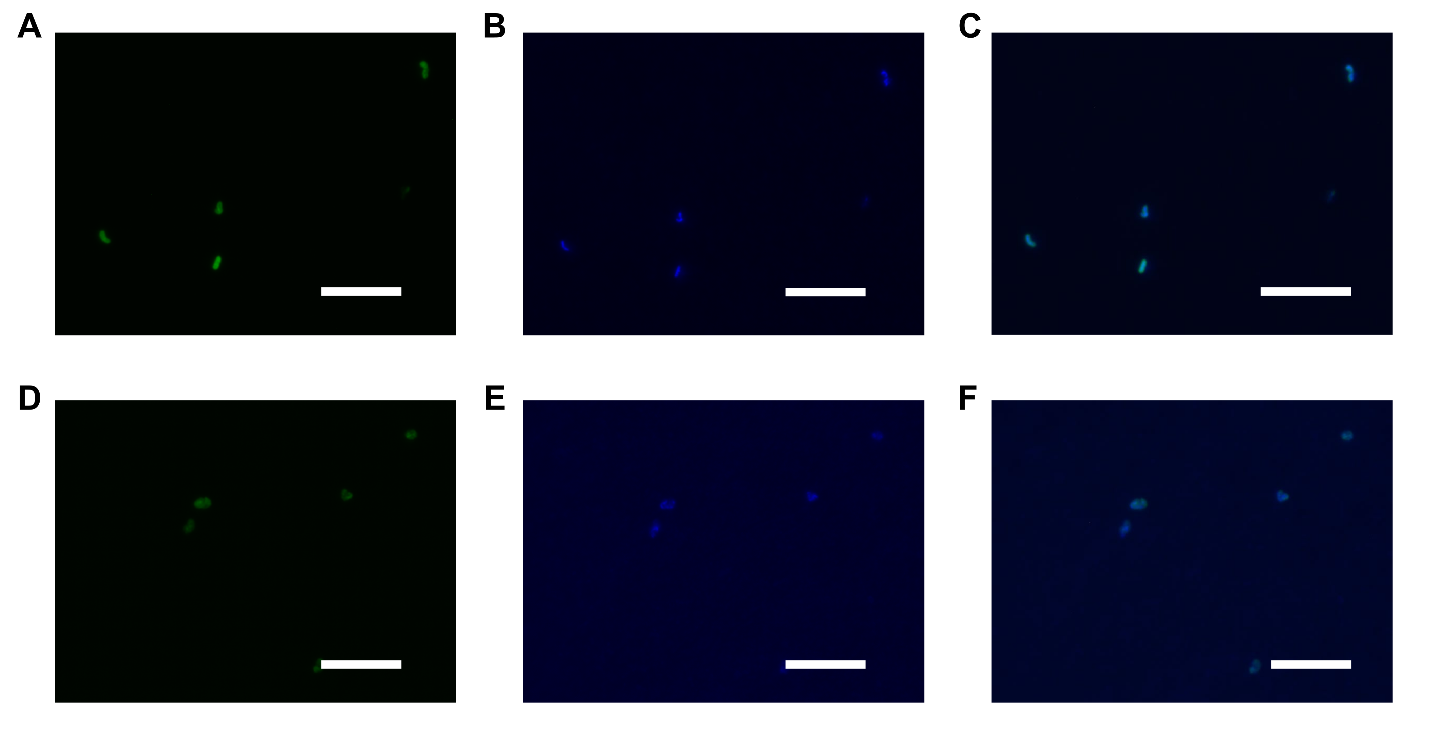


**Supplementary Figure 5:** Fluorescence microscopy images of *Polaribacter sp.* KT25b grown with glucose (**A-C**) and laminarin (**D-F**) after FISH and FACS in the green channel (**A**, **D**), ultraviolet channel (**B**, **E**) and merged. FISH probe signals are green and DAPI-stained cells are blue. Scalebar: 10 µm (applies to all panels).


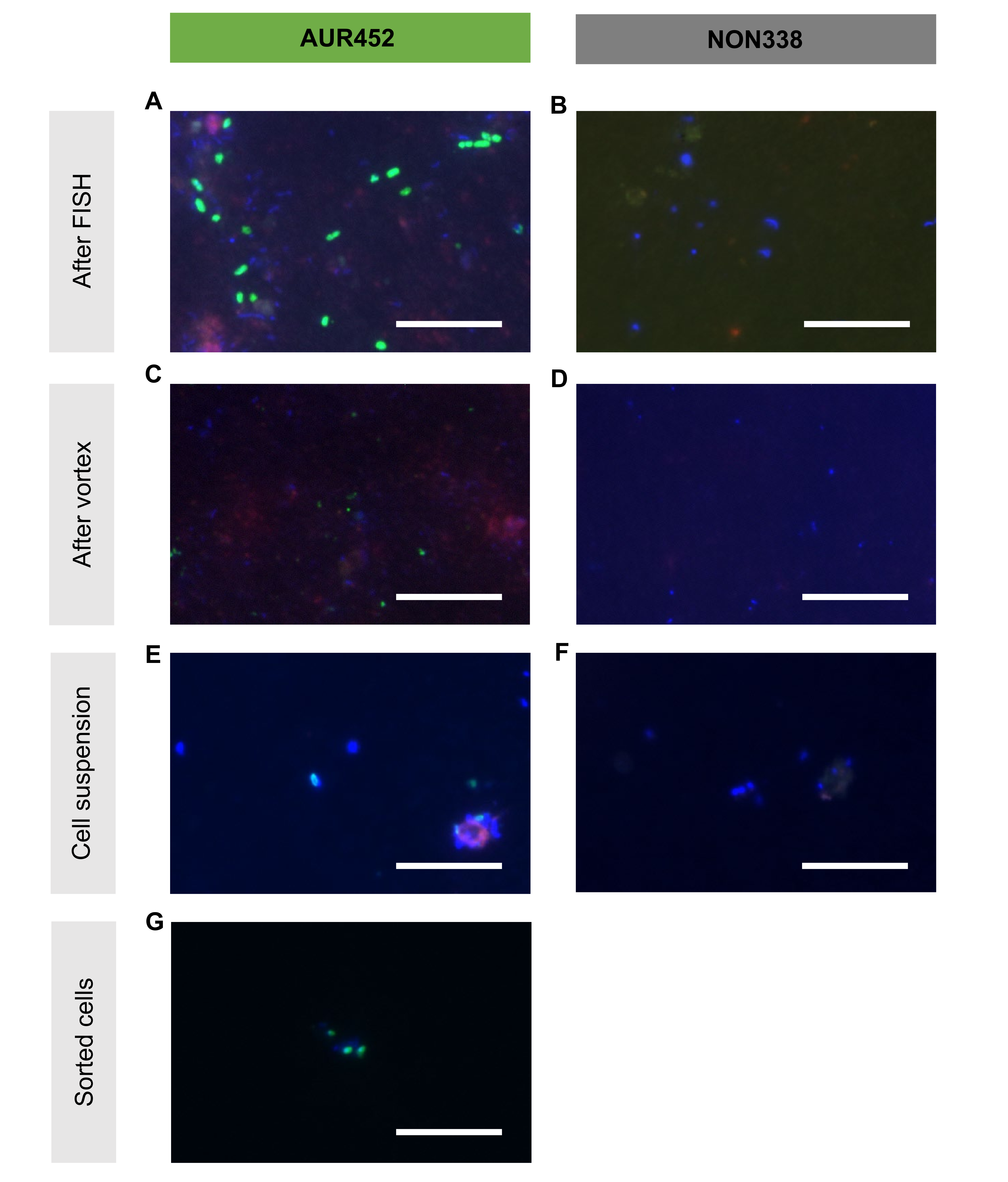


**Supplementary Figure 6:** Fluorescence microscopy images of bacterial cells from environmental samples at various steps of the FISH-FACS workflow for AUR452 (**A**, **C**, **E**, **G**) and NON338 (**B**, **D**, **F**) pieces before vortexing (**A**, **B**), after vortexing (**C**, **D**), and cell suspensions before (**E**, **F**), and after sorting (**G**). Green signals are FISH signals, blue signals are DAPI stained, red is natural autofluorescence. Scalebar: 10 µm (applies to all panels).

# **
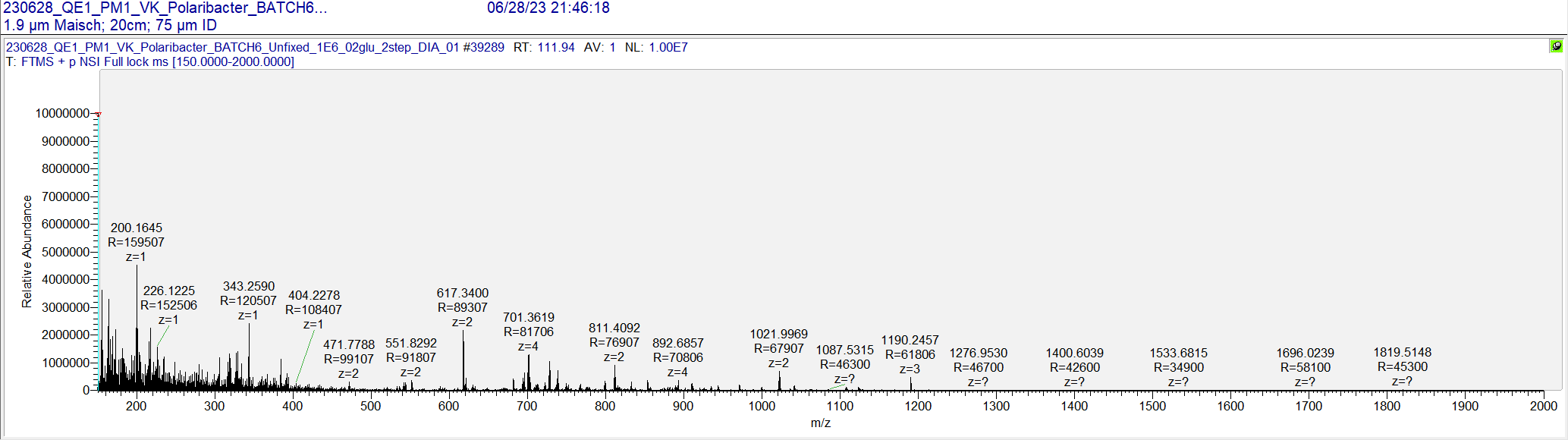
A**


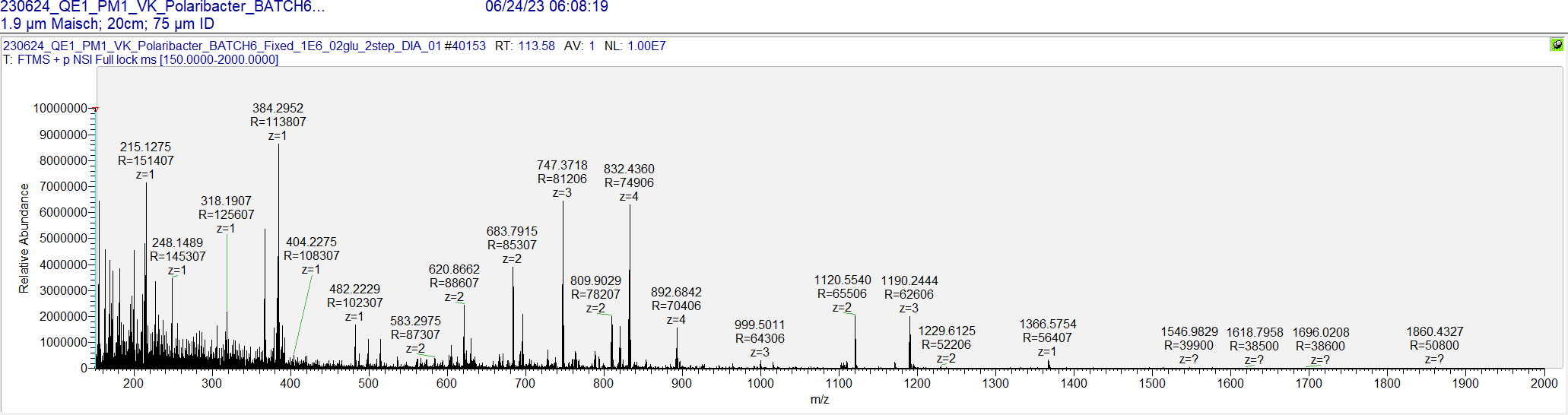
**B**

**C**


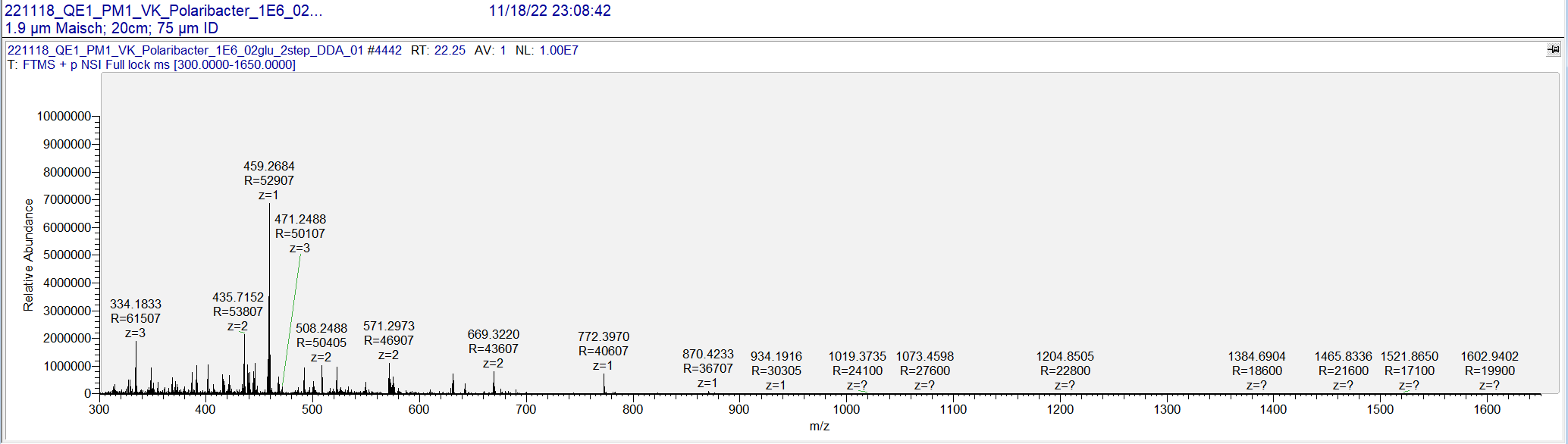

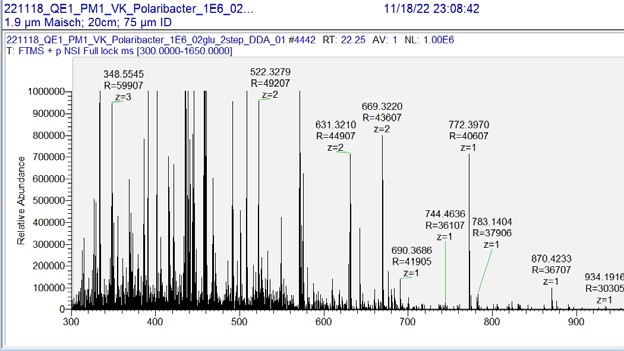


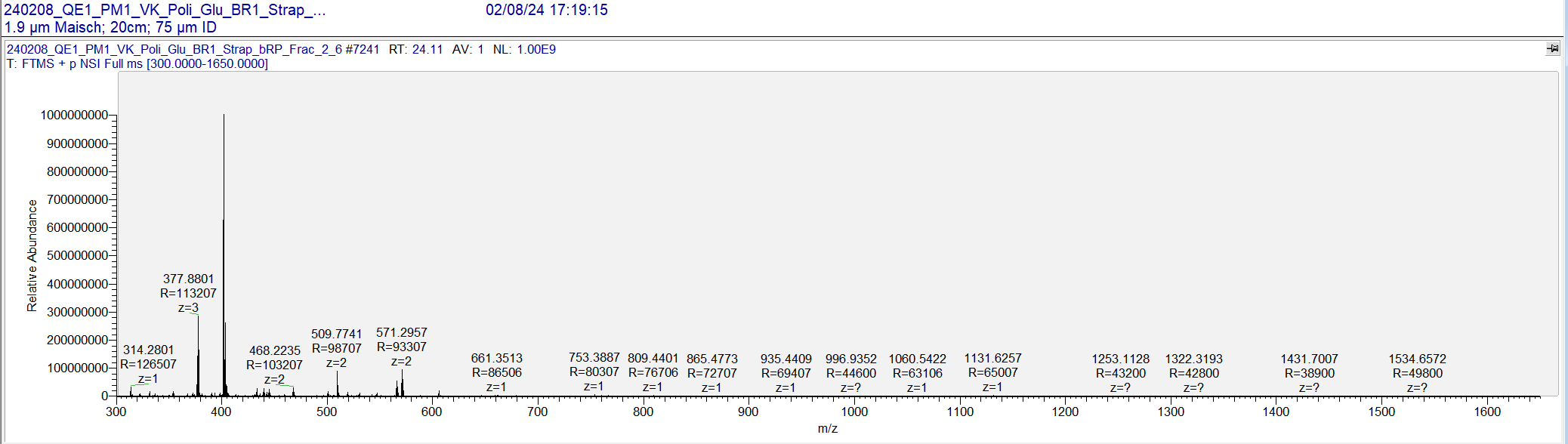
**D**


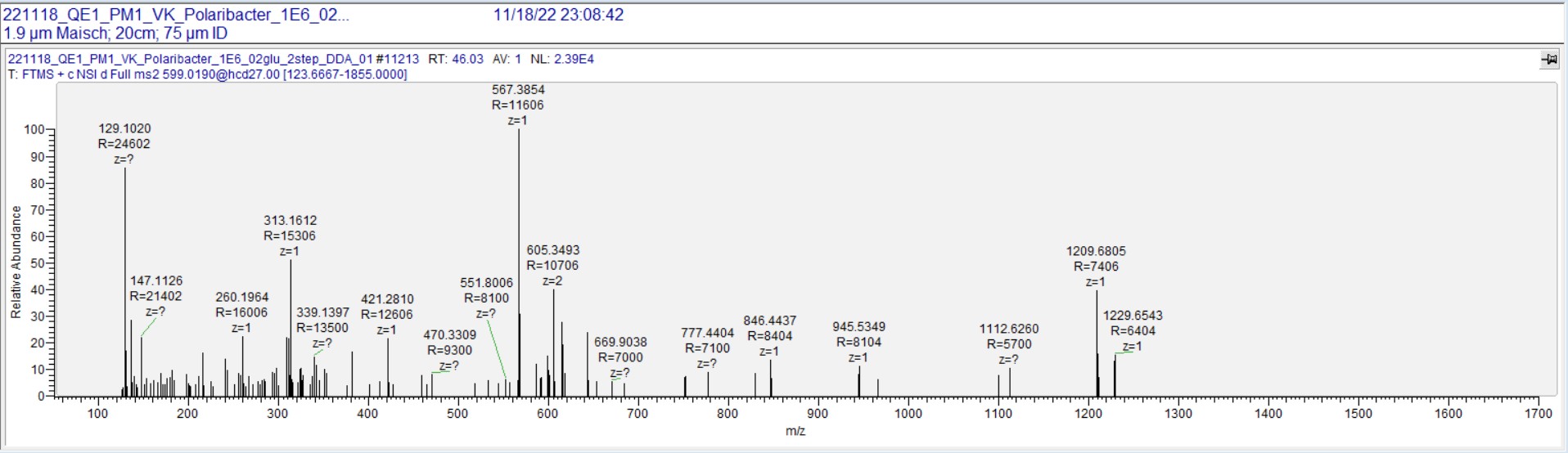
**E**


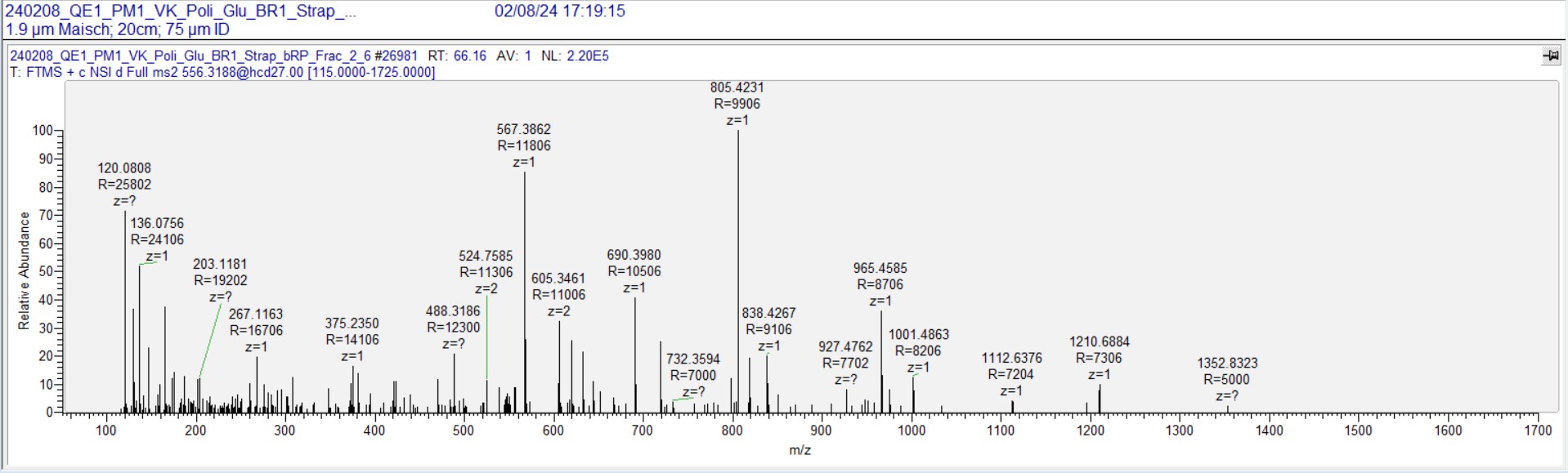
**F**


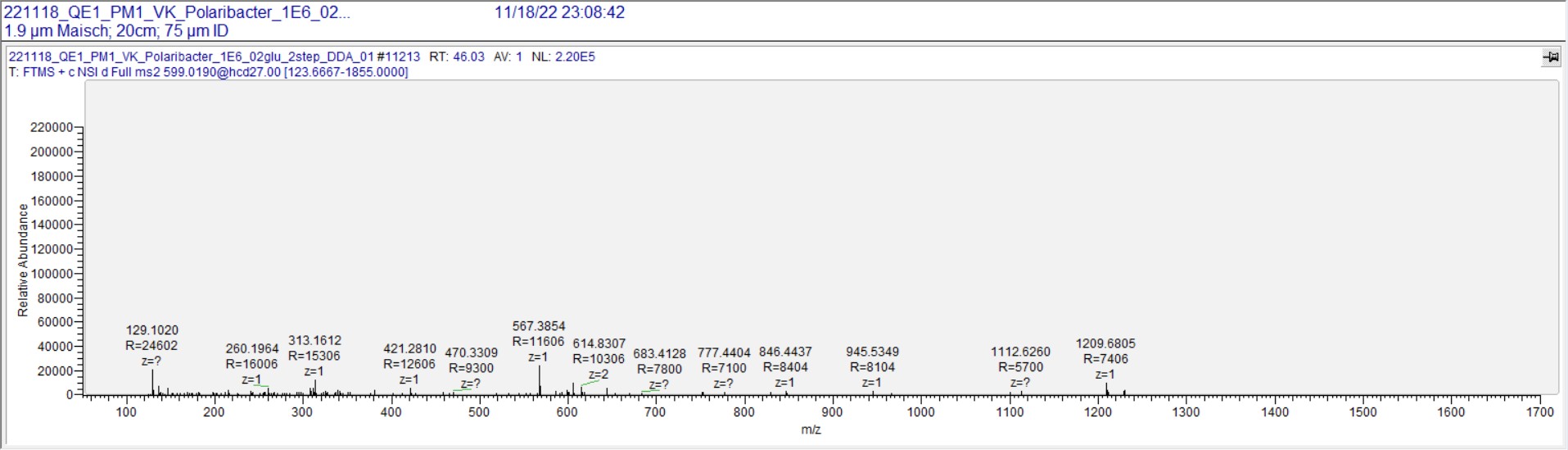
**G**


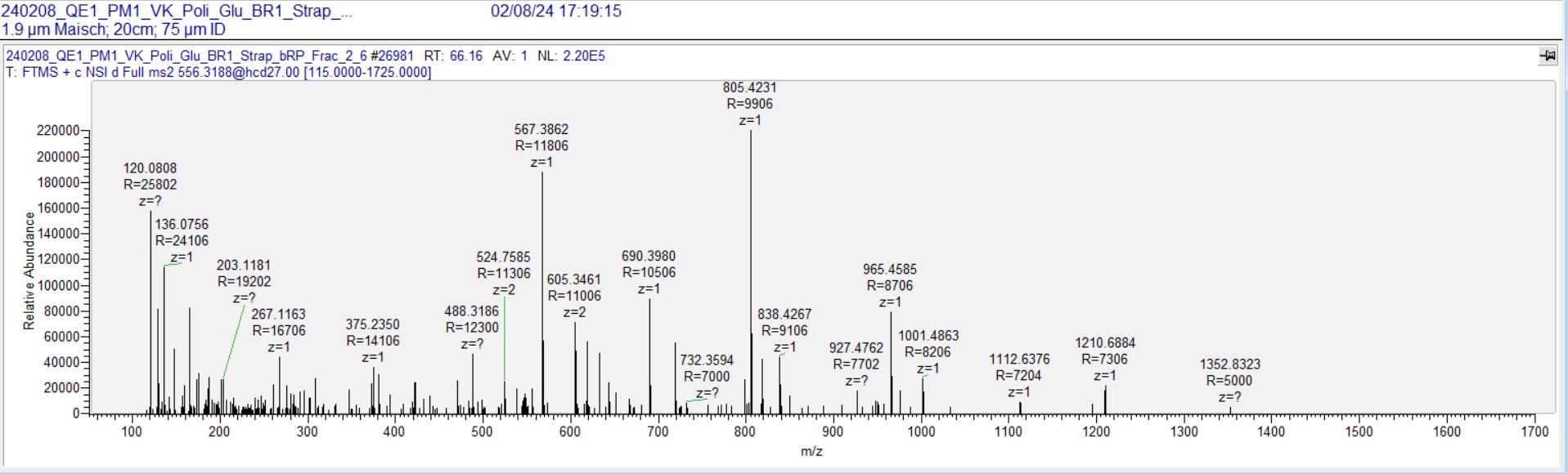
**H**

**Supplementary Fig. 7:** Example mass spectra obtained from DIA (data-independent acquisition) measurements of low cell number samples and from DDA (data-dependent acquisition) measurements of high-biomass S-Trap samples (50 µg digested protein) and preliminary experiments with low cell number (unfixed 1 × 10⁶ *Polaribacter* sp. KT25b cells). The MS1 spectra (DIA) of 1 × 10⁶ *Polaribacter* sp. KT25b cells, both unfixed (**A**) and fixed (**B**), display high peak densities and charged peptide ions. Panels **C-H** present MS1 and MS2 spectra from both low cell number DDA-acquired and the high biomass S-Trap DDA-acquired samples. The MS1 spectra from the low cell number DDA-acquired sample (**C**) show reduced peak intensity compared to high biomass S-Trap sample (**D**), though the inset in (**C**) highlights peak densities comparable to those seen in DIA spectra of low cell number samples. The MS2 spectra of the peptide fragment LSEKPPADYVPPIIK show approximately one order of magnitude increase in peak intensity in the S-Trap DDA-acquired sample (**F**) relative to the low cell number DDA-acquired sample (**E**), with each spectra independently scaled on the y-axis for relative abundance. When both spectra are visualised using the same y-axis scale, a decrease in the relative abundance of this peptide fragment in the low cell number DDA-acquired sample (**G**) compared to the S-Trap DDA-acquired sample (**H**) is evident.

**
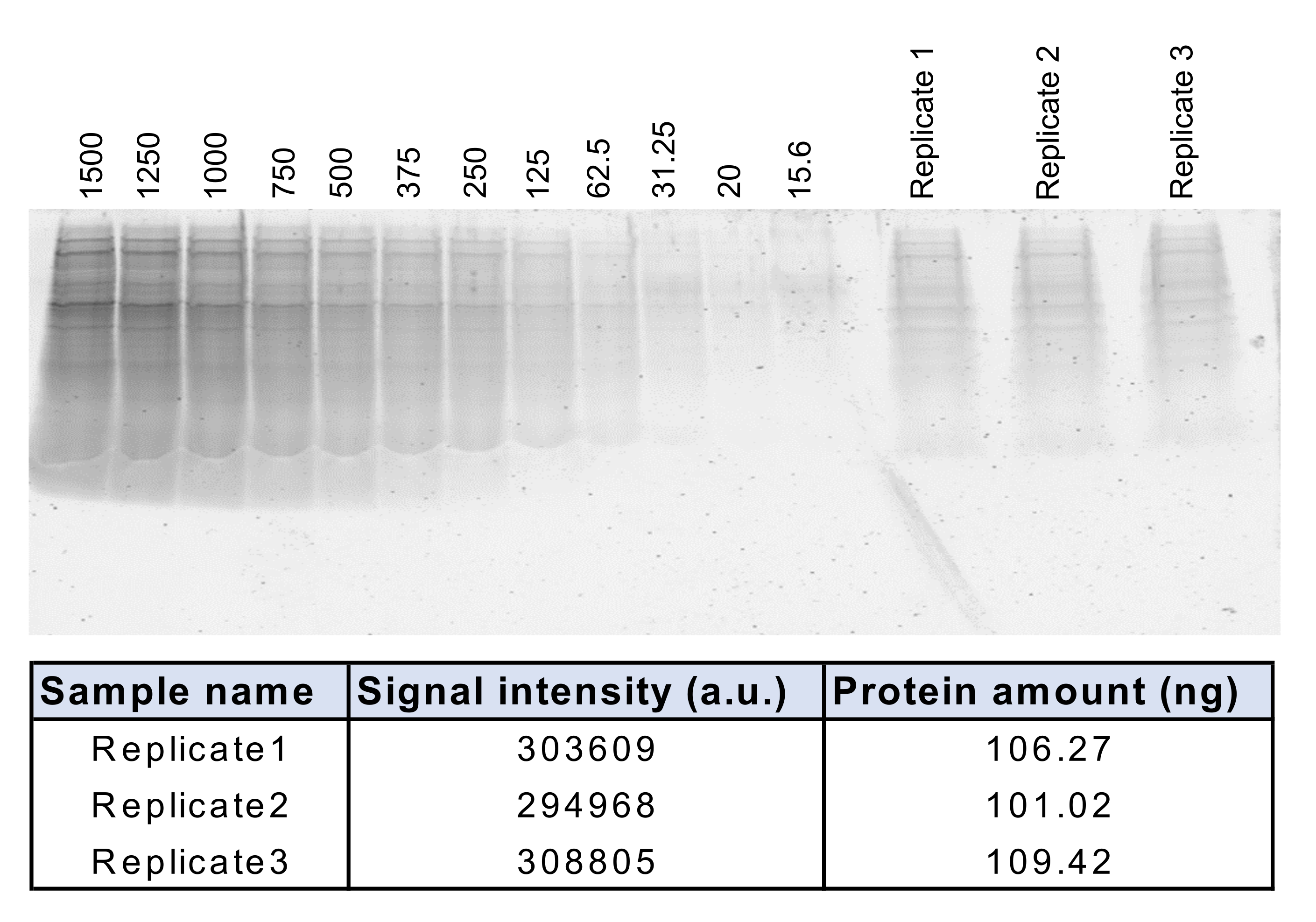
**

**Supplementary Figure 8:** Protein estimation was performed using Flamingo gel stain (Bio-Rad) on preliminary samples derived from 1x10^6^ *Polaribacter* sp. KT25b cells (replicates 1-3). A standard protein curve was generated using a range of known protein amount (1500–15.6 ng) prepared from a high-biomass protein extract of *Polaribacter* sp. KT25b cells. By correlating the fluorescence signal intensity (a.u.) for each sample to the standard curve, we estimated the protein amount corresponding to defined number of cells (1x10^6^). This provided an estimation of starting protein amount for downstream proteomic sample preparation.


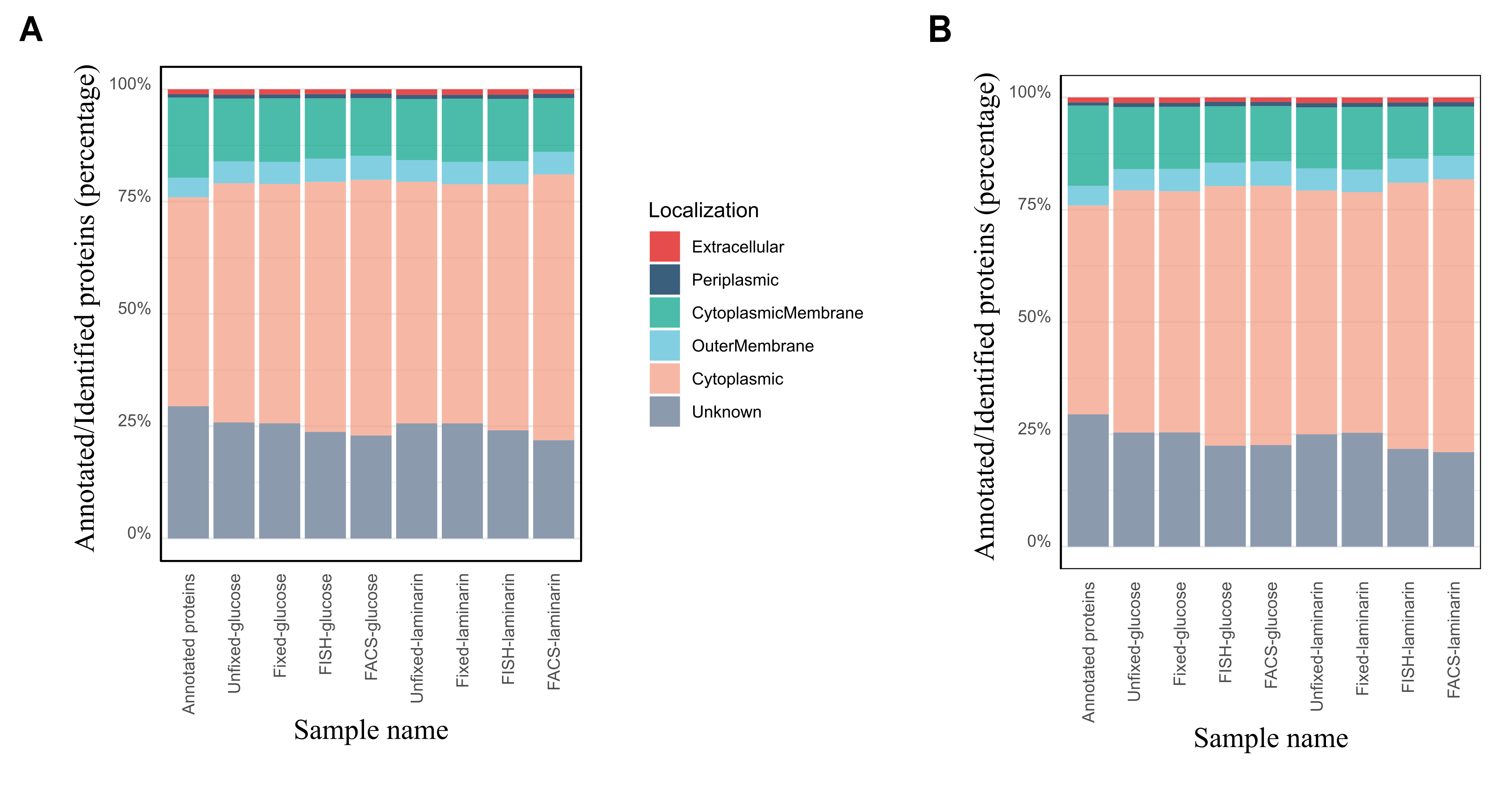


**Supplementary Figure 9:** Subcellular based localisation based on in silico prediction tool PSORTb (v3.0.3) in (**A**) 1x10^6^ and (**B**) 5x10^5^ *Polaribacter* sp. KT25b glucose and laminarin grown cells.


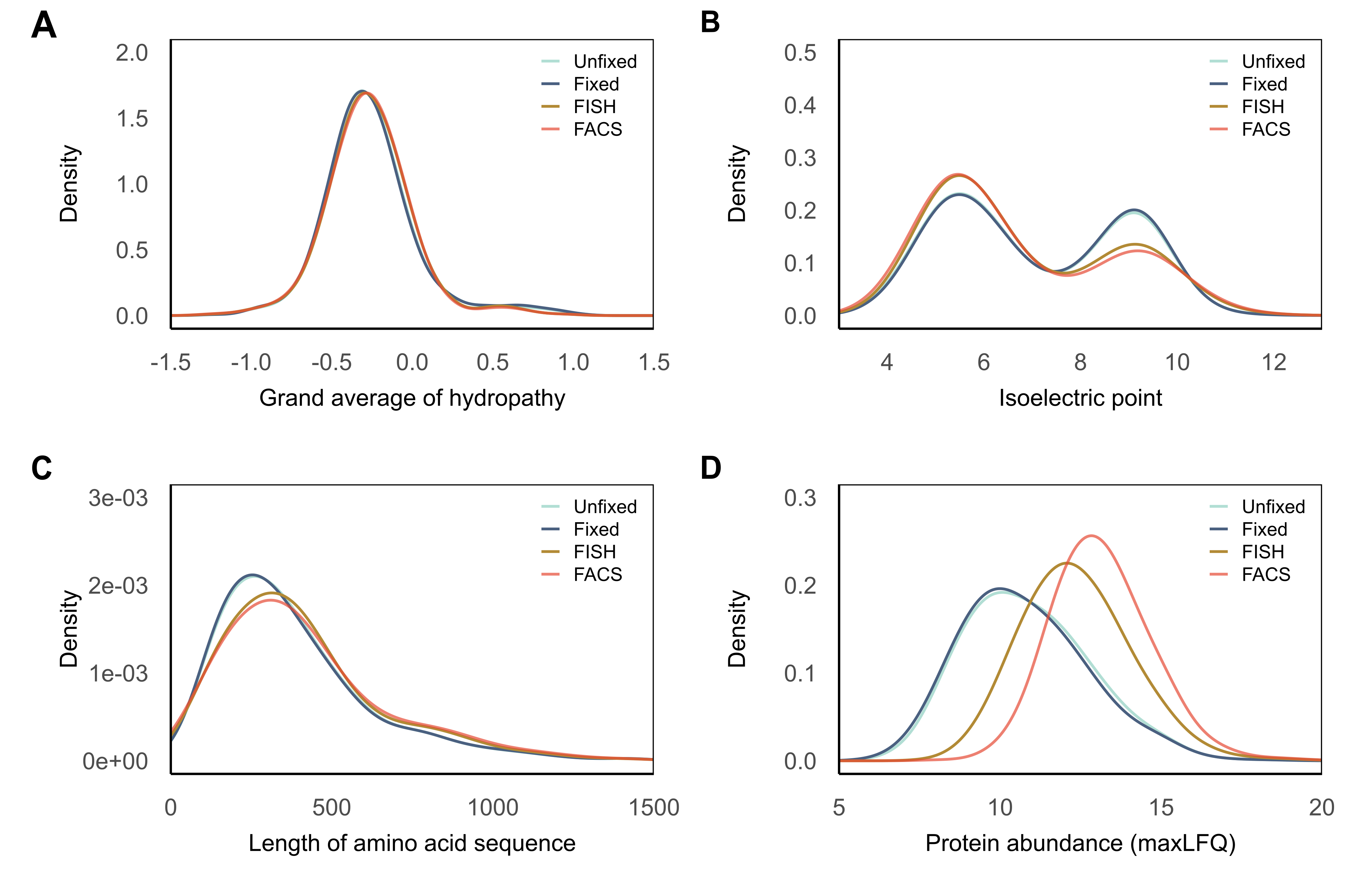


**Supplementary Figure 10:** Physicochemical properties of proteins identified in five out of five sample replicates in a density plot of 5x10^5^ *Polaribacter* sp. KT25b cells grown in either glucose or laminarin as a carbon source. Individual lines represent the sequential steps in the protocol. The distribution of identified proteins based on (**A**) the grand average of hydropathy, (**B**) the isoelectric point and (**C**) the length of the amino acid sequence and (**D**) protein quantification values (maxLFQ).


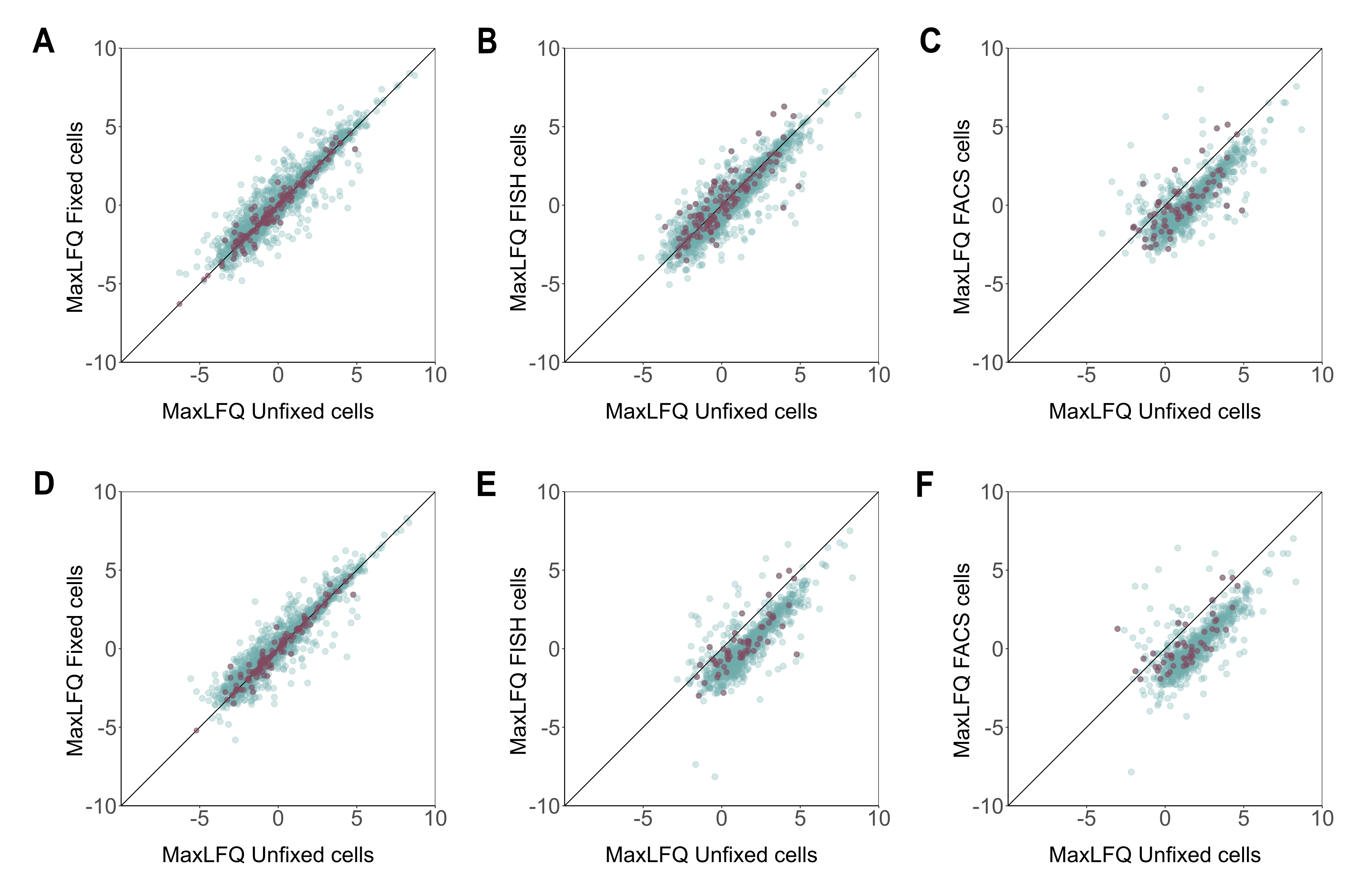


**Supplementary Figure 11:** Correlation between quantification values (maxLFQ) of identified proteins. Data represents proteins quantified from 1x10^6^ *Polaribacter* sp. KT25b cells (**A**, **B**, **C**) and 5x10^5^ cells (**d**, **e**, **f**) grown with laminarin. Highlighted proteins (in red) are carbohydrate-active enzymes and TonB-dependent transporters.

**
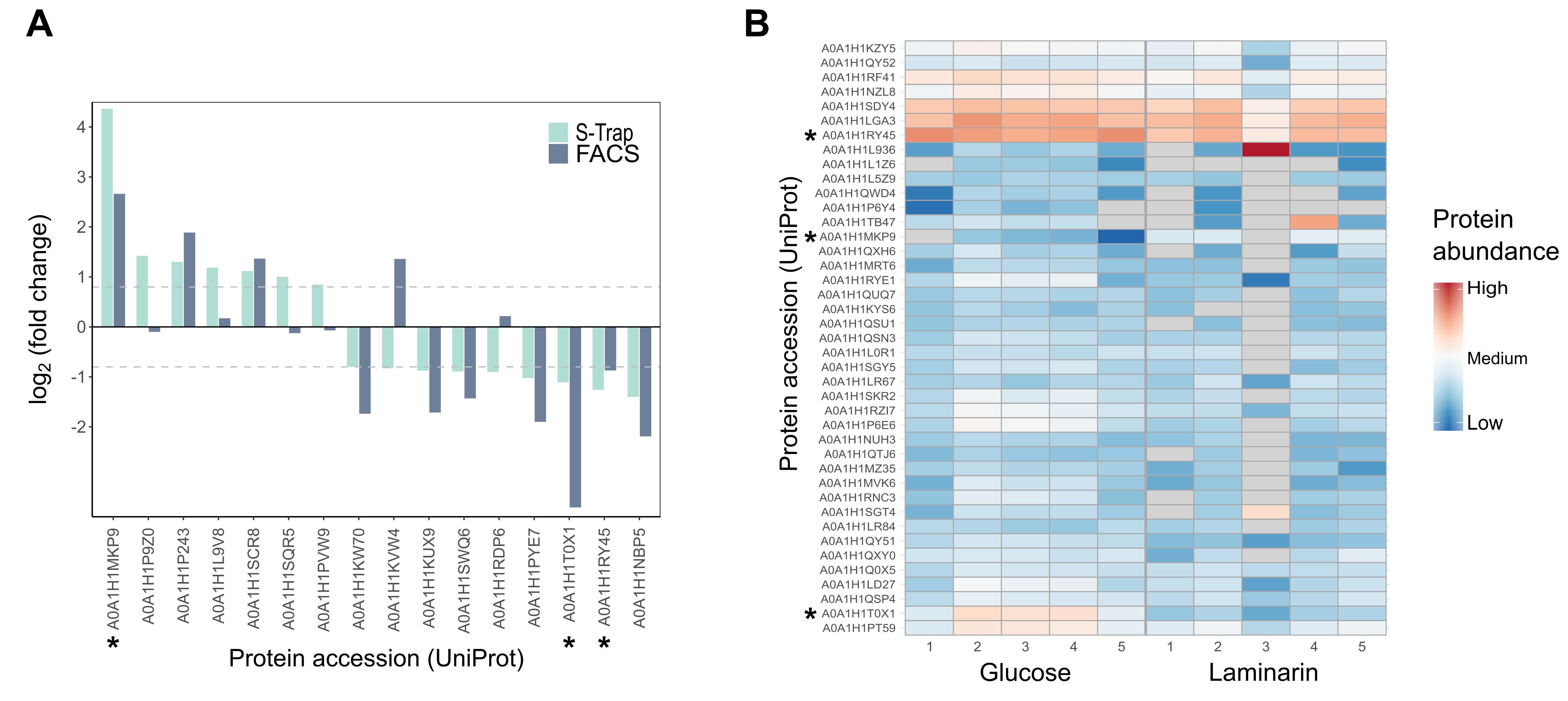
**

**Supplementary Figure 12:** Protein abundance analysis based on label-free quantification (maxLFQ) values. (**A**) Comparison of fold change for significantly different abundant proteins in samples prepared from S-Trap and FISH-FACS proteomics (5x10^5^ cells) workflow. (**B**) Heatmap visualisation of protein abundance of carbohydrate-active enzymes and TonB-dependent transporters in samples prepared from 5x10^5^ sorted *Polaribacter* sp. KT25B cells. * Proteins of interest discussed in this study.

**
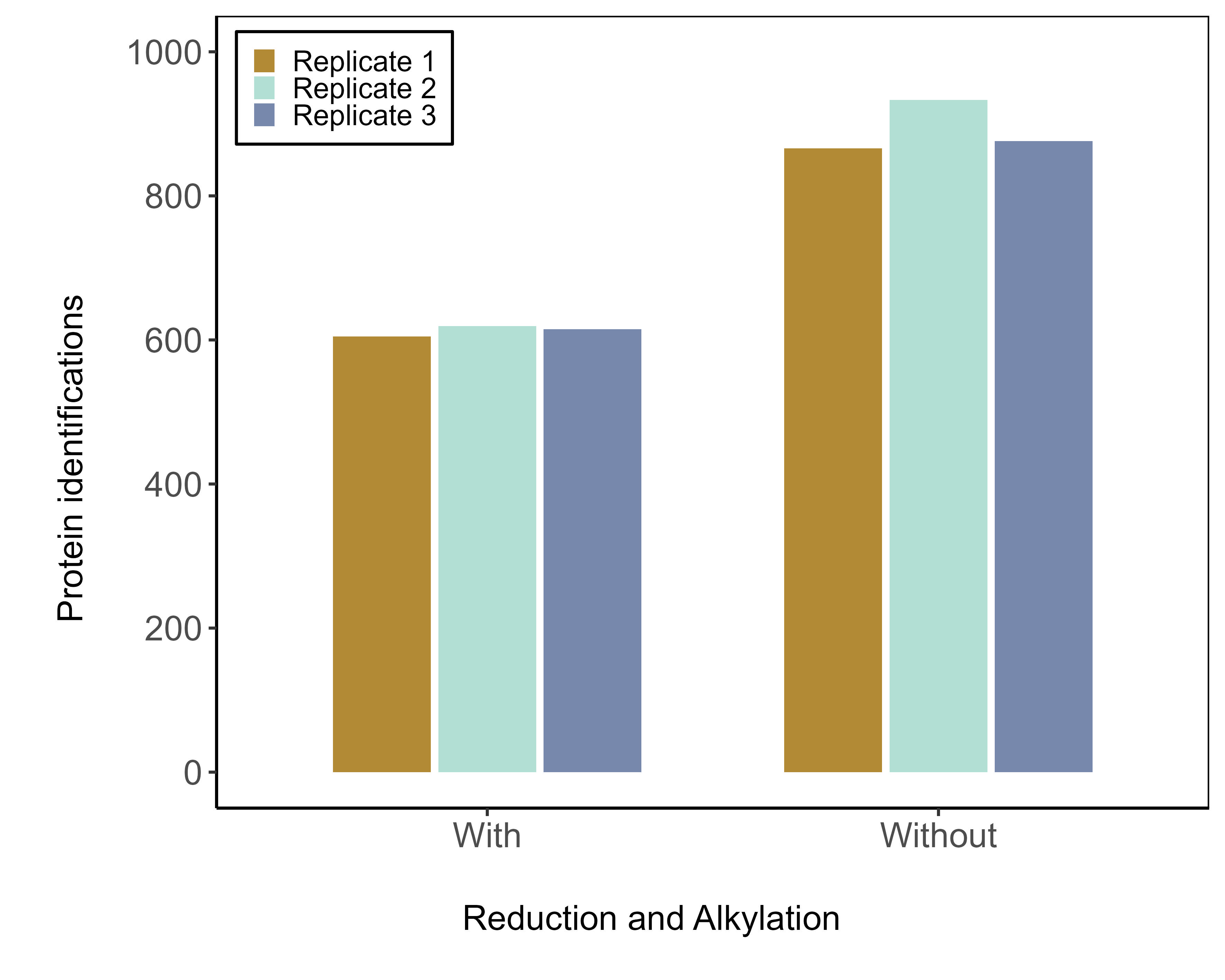
**

**Supplementary Figure 13:** Protein identifications from preliminary direct digestion experiments performed on 1x10^6^ *Polaribacter* sp. KT25b cells, with or without reduction and alkylation step using 250mM Tris (2-carboxyethyl) phosphine and 500mM iodoacetamide.

**
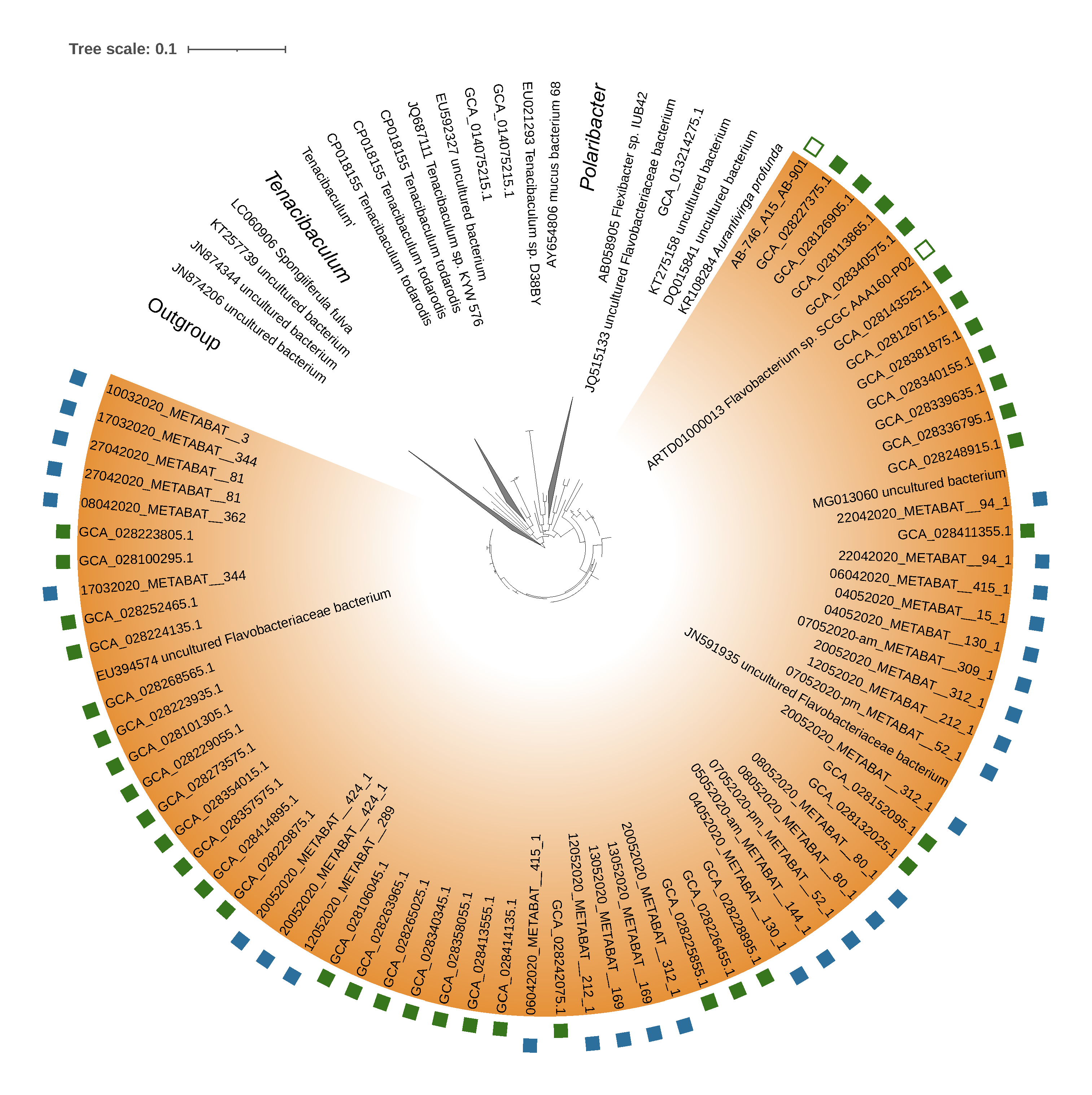
**

**Supplementary Figure 14:** Consensus phylogenetic tree of 16S rRNA gene sequences extracted from uncultured genomes in the GTDB genus SCGC-AAA160-P02. Bold type face denotes that the genome is found in GTDB v. 214. Blue squares signify the sequence originates from the Helgoland 2020 sampling campaign. Green squares signify the sequence is a SAG; green filled squares derive from reference [1]. SILVA reference sequences are prefixed by the SILVA accession code. The genome from source [2] is labelled with the label AB-746_A15_AB-901, as it lacks an NCBI genome accession number.

**Generation of 16S tree and AUR452 probe coverage check**

A 16S rRNA tree was constructed in the program ARB [3] in order to verify the theoretical specificity of the oligonucleotide probe AUR452 with competitors AUR452-c1 and AUR452-c2 (**Supplementary Table 1**). 191 culture-independent genomes of *Aurantivirga* were identified for rRNA gene extraction. The collection includes all genomes with the genome classification SCGC-AAA160-P02 from GTDB (v. 214 release 2023) [4] (n=21), MAGs generated from the sampling campaigns at Helgoland from the years 2010-2020 (n=110, of which 4 are included in GTDB v.214) [5-7] and SAGs collected from the coastal sampling sites at Boothbay Harbor (n=63) [1] and Saanich Inlet (n=1) [2]. 16S rRNA gene sequences were extracted from all genomes using Barrnap v.0.9 [8] and imported into the SILVA non-redundant reference database (SSU ref NR99 v.138.1) [9] and aligned using the SINA aligner [10]. A preliminary tree was generated by using the “Quick Add (Parsimony)” function using the filter “Pos. Var: Bac” with the ‘ignored values’ set to “.=-0123456”, which adds aligned user sequences the reference tree. Using the preliminary tree, sequences located outside of the family *Flavobacteriaceae* or long branching were removed after manual inspection of the sequence alignment. In order to reduce unnecessary tree calculations for de-novo tree generation, duplicate sequences were excluded by de-selecting all but one sequence within a cluster of sequences with 0 branch length. The checked sequences and reference sequences belonging to the genera *Tenacibaculum*, *Polaribacter*, and *Aurantivirga* were selected for tree calculation (n=363). Neighbour joining trees based on Jukes-Cantor distance matrices were calculated with and without a *Bacteroides*-specific 30% positional variability filter. Maximum likelihood trees were calculated with and without a *Bacteroides*-specific 30% positional variability filter using RAxML 8 using 10 searches. A consensus tree from the four trees was generated and refined. Finally, the duplicate sequences were added to the tree using the “Quick add parsimony” using the filter “Pos. Var: Bac” but with ‘ignored values’ set to “. =-0” from the preliminary tree building step. Exact probe matches to AUR452 minus exact matches to the competitors, were determined in ARB and the resulting tree was visualised with the probe match information in the interactive tree of life (iTOL) [11].

**Supplementary Table 1:** Oligonucleotide probes used in this publication.

| **Probe Name** | **Sequence** | **Fluorophore** | **References** |
| --- | --- | --- | --- |
| CF319a | UGGTCCGTGTCTCAGTAC | 4x Atto 488 | 12 |
| NON338 | ACT CCT ACG GGA GGC AGC | 4x Atto 488 | 13 |
| AUR452 | AGC ACC TAC ACG TAG GTG TGT T | 4x Atto 488 | 7 |
| AUR452-c1 | CAGACACCTACACGTAGGTGCGTT |  | 14 |
| AUR452-c2 | AGCACCTACACGTAGGTGTGGTTC |  | 14 |

**Supplementary Table 2:** Laser, photomultiplier tube (PMT), optical filters configured on the BD Influx Mariner FACS instrument, and axis labels found in **Supplementary Fig. 3, 4**.

| **Laser** | **Wavelength** | **PMT** | **filter** | **Axis label (SF. 4+5)** |
| --- | --- | --- | --- | --- |
| **laser 1** | **488** | 1 | 488/10 |  |
| **laser 1** | **488** | 2 | 488/10 | Forward scatter |
| **laser 1** | **488** | 3 | 530/40 | Green fluorescence |
| **laser 1** | **488** | 4 | 580/30 | Yellow fluorescence |
| **laser 1** | **488** | 5 | 610/20 |  |
| **laser 1** | **488** | 6 | 670/30 |  |
| **laser 2** | **355** | 7 | 460/50 |  |
| **laser 2** | **355** | 8 | 530/40 |  |
| **laser 2** | **355** | 9 | 650 LP |  |
| **laser 3** | **640** | 10 | 570 LP | Red fluorescence |
| **laser 4** | **561** | 11 | 615/24 |  |
| **laser 4** | **561** | 12 | 585/29 |  |

**Supplementary Table 3:** Protein report file from low biomass samples from *Polaribacter* sp. KT25b and high biomass samples (S-Trap protocol).

**Supplementary Table 4:** Protein and peptide report file from bacterial cells (*Auranitvirga* spp.) from environmental samples.

**Supplementary Table 5:** Protein and peptide report file from unsorted metaproteomics samples.

**Supplementary Table 6:** Sample overview of all *.raw files submitted to the PRIDE repository (ID: PXD057908).

**The Supplementary Table 3, 4, 5, and 6 are available as stand-alone files.**

**References**

1. Munson-McGee JH, Lindsay MR, Sintes E, Brown JM, D’Angelo T, Brown J, *et al.* Decoupling of respiration rates and abundance in marine prokaryoplankton. *Nature,* 2022;**612**:764-770. https://doi.org/10.1038/s41586-022-05505-3.
2. Anstett J, Plominsky AM, DeLong EF, Kiesser A, Jürgens K, Morgan-Lang C, *et al.* A compendium of bacterial and archaeal single-cell amplified genomes from oxygen deficient marine waters. *Scientific data*, 2023;**10**:332. https://doi.org/10.1038/s41597-023-02222-y.
3. Ludwing W, Strunk O, Westram R, Richter L, Meier H, Yadhukumar A, *et al.* ARB: a software environment for sequence data. *Nucleic acids research*, 2004;**32**:1363-1371. https://doi.org/10.1093/nar/gkh293.
4. Parks DH, Chuvochina M, Rinke C, Mussig AJ, Chaumeil PA, Hugenholtz P. GTDB: an ongoing census of bacterial and archaeal diversity through a phylogenetically consistent, rank normalized and complete genome-based taxonomy. *Nucleic acids research* 2022; **50**: 785-794. https://doi.org/10.1093/nar/gkab776.
5. Teeling H, Fuchs BM, Becher D, Klockow C, Gardebrecht A, Benneke CM, *et al.* Substrate-controlled succession of marine bacterioplankton populations induced by a phytoplankton bloom. Science, 2012;**336**:608–611. https://doi.org/10.1126/science.1218344.
6. Teeling H, Fuchs BM, Benneke CM, Krüger K, Chafee M, Kappelmann L, *et al.* Recurring patterns in bacterioplankton dynamics during coastal spring algae blooms. elife, 2016;**5**:e11888. https://doi.org/10.7554/eLife.11888.
7. Sidhu C, Kirstein IV, Meunier CL, Rick J, Fofonova V, Wiltshire KH, *et al.* Dissolved storage glycans shaped the community composition of abundant bacterioplankton clades during a North Sea spring phytoplankton bloom. Microbiome, 2023;**11**:77. https://doi.org/10.1186/s40168-023-01517-x.
8. Seemann T. Barrnap 0.9: rapid ribosomal RNA prediction. https://github.com/tseemann/barrnap.
9. Quast C, Pruesse E, Yilmaz P, Gerken J, Schweer T, Yarza P, *et al.* The SILVA ribosomal RNA gene database project: improved data processing and web-based tools. *Nucleic acids research*, 2012;**41**:590-596. https://doi.org/10.1093/nar/gks1219.
10. Pruesse E, Peplies J, Glöckner FO. SINA: Accurate high-throughput multiple sequence alignment of ribosomal RNA genes. *Bioinformatics*, 2012;**28**:1823-1829. https://doi.org/10.1093/bioinformatics/bts252.
11. Letunic I, Bork P. Interactive Tree of Life (iTOL) v6: recent updates to the phylogenetic tree display and annotation tool. *Nucleic acids research*, 2024;**52**:78-82. https://doi.org/10.1093/nar/gkae268.
12. Manz W, Amann R, Ludwig W, Vancanneyt M, Schleifer KH. Application of a suite of 16s rRNA-specific oligonucleotide probes designed to investigate bacteria of the phylum cytophaga-flavobacter-bacteroides in the natural environment. *Microbiology*, 1996;**142**:1097–1098. https://doi.org/10.1099/13500872-142-5-1097.
13. Manz W, Amann R, Ludwig W, Wagner M, Schleifer KH. Phylogenetic Oligodeoxynucleotide Probes for the Major Subclasses of Proteobacteria: Problems and Solutions. *Syst Appl Microbiol*, 1992;**15**:593–600. https://doi.org/10.1016/S0723-2020(11)80121-9.
14. Brüwer JD, Orellana LH, Sidhu C, Klip HC, Meunier CL, Boersma M, *et al.* In situ cell division and mortality rates of SAR11, SAR86, Bacteroidetes, and Aurantivirga during phytoplankton blooms reveal differences in population controls. *Msystems*, 2023;**8**:e01287-22. doi:10.1128/msystems.01287-22.
